# Metagenomic insights into microbial community, functional annotation, and antibiotic resistance genes in Himalayan Brahmaputra River sediment, India

**DOI:** 10.3389/fmicb.2024.1426463

**Published:** 2024-11-20

**Authors:** Niti Sharma, Basanta Kumar Das, Birendra Kumar Bhattacharjya, Aparna Chaudhari, Bijay Kumar Behera, Annam Pavan Kumar, Hirak Jyoti Chakraborty

**Affiliations:** ^1^ICAR-Central Inland Fisheries Research Institute, Regional Centre, Guwahati, Assam, India; ^2^ICAR-Central Inland Fisheries Research Institute, Kolkata, India; ^3^ICAR-Central Institute of Fisheries Education, Mumbai, Maharashtra, India

**Keywords:** river sediment, Brahmaputra River, metagenomics, microbial communities, antibiotic resistance genes, anthropogenic activities

## Abstract

**Introduction:**

The Brahmaputra, a major transboundary river of the Himalayas flowing predominantly through Northeast India, particularly Assam, is increasingly endangered by contamination due to rapid urbanization and anthropogenic pressures. These environmental changes pose significant risks at the microbial level, affecting nutrient cycling and productivity, and thereby impacting river ecosystem health. The next-generation sequencing technology using a metagenomics approach has revolutionized our understanding of the microbiome and its critical role in various aquatic environments.

**Methods:**

The present study aimed to investigate the structure of the bacterial community and its functional potentials within the sediments of the Brahmaputra River, India, using high-throughput shotgun metagenomics. Additionally, this study sought to explore the presence of antimicrobial resistance genes in the river’s sediment.

**Results and discussion:**

Shotgun metagenomics revealed a diverse bacterial community comprising 31 phyla, 52 classes, 291 families, 1,016 genera, and 3,630 species. Dominant phyla included Pseudomonadota (62.47–83.48%), Actinobacteria (11.10–24.89%), Bacteroidetes (0.97–3.82%), Firmicutes (0.54–3.94%), Cyanobacteria (0.14–1.70%), and Planctomycetes (0.30–0.78%). Functional profiling highlighted significant involvement in energy metabolism, amino acid and central carbon metabolism, stress response, and degradation pathways, emphasizing the microbial community’s role in ecosystem functioning and resilience. Notably, 50 types of antibiotic resistance genes (ARGs) were detected, with resistance profiles spanning multidrug, aminoglycoside, *β*-lactam, fluoroquinolone, rifampicin, sulfonamide, and tetracycline classes. Network analysis underscored the intricate relationships among ARG subtypes, suggesting potential mechanisms of resistance propagation. Furthermore, plasmid-related genes and 185 virulence factor genes (VFGs) were identified, indicating additional layers of microbial adaptation and potential pathogenicity within the river sediments. This comprehensive microbial and functional profiling of the Brahmaputra’s sediment metagenome provides crucial insights into microbial diversity, resistance potential, and ecological functions, offering a foundation for informed management and mitigation strategies to preserve river health and mitigate pollution impacts.

## Introduction

The Himalayan Brahmaputra River is one of the world’s largest transboundary river systems, flowing through India (33.6%), China (50.5%), Bangladesh (8.1%), and Bhutan (7.8%) originating from the massive glacier mass in the southern Tibetan Kailash range (Singh et al., 2004). In India, the river flows for 916 km, entering through Northeast India. A major portion, approximately 640 km, flows through Assam, where the river gets the name Brahmaputra, flowing along the southern and northern banks (Datta and Singh, 2004). Large water volumes, considerable silt, rapid bed aggradations, continuous changes in channel morphology, and bank line erosion are some of the characteristics that define the mighty river (NDMA, 2012). The river is the fifth largest in terms of flow and serves as the primary water source for over 130 million people, providing essential space and resources (Mahanta et al., 2014; Ray et al., 2015). The river system is experiencing increased stress as a result of rapid industrialization and urbanization occurring in and around the river (Bhattacharjya et al., 2017). The river flowing through urban areas are severely endangered and contaminated due to anthropogenic activities and inadequate waste management. In recent years, pollution levels in the Brahmaputra River have increased due to population growth and increased water utility demands. With an average annual discharge of 591 km^3^/year, the river produces approximately 179 million liters of sewage per day. Between 2006 and 2019, water turbidity increased significantly, and microplastic pollution reached extremely high levels during 2018–2019. These trends signal serious concerns regarding the impacts of pollution on both the environment and human health (Bora and Goswami, 2017; Rakhecha, 2020; Barbulescu et al., 2021).

Microbes in river ecosystems are crucial for recycling nutrients, mineralizing organic matter, maintaining the ecosystem quality, facilitating energy flow in the food chain, degrading heavy metals, and many other beneficial properties (Findlay, 2010; Madsen, 2011; Liu et al., 2012). River sediments are the primary site where both pollutants and microbes adhere and accumulate; hence, the abundance, diversity, and stability of microbes are greatly impacted by numerous anthropogenic pollutants that are present (Devarajan et al., 2015; Saxena et al., 2015; Beattie et al., 2020). The impact of anthropogenic pollution on the microbial population composition can help predict the health and functionality of the ecosystem (Galand et al., 2016; Santillan et al., 2019). However, it is challenging to completely comprehend the structure, function, and interactions in such complex aquatic systems using culture-based techniques. Metagenomics has emerged as the ideal technique for the comprehensive study of the taxonomic and functional profile of microbial communities. Whole-genome metagenomic sequencing has revealed an understanding of the taxonomic diversity of freshwater and marine microbial species worldwide (Vogel et al., 2009; Toyama et al., 2016).

Metagenomics sequencing has emerged as a handy tool for exploring the antibiotic resistance profiles of microbes in the aquatic ecosystem (Xu et al., 2018; Li et al., 2020; Das et al., 2020). The aquatic environment represents a reservoir of resistance bacteria or antibiotic resistance genes (ARGs) that could be transmissible to animals and humans (Wang et al., 2020; Zhuang et al., 2021). Aquatic systems significantly impact the accumulation and spread of antibiotic resistance bacteria and ARGs in sediments and freshwater (Pham et al., 2018; Zhao et al., 2018). Mobile genetic elements (MGEs) such as plasmids, which function as a vector of ARG transfer and shape ARG patterns in microbial communities, mediate the horizontal gene transfer of ARGs (Zhang et al., 2011; Mencía-Ares et al., 2020). In recent years, the presence of antimicrobial resistance and ARGs has been reported in various aquatic ecosystems, posing a risk to human health (Taylor et al., 2011; Reddy and Dubey, 2019). Similarly, virulence factor genes (VFGs) of pathogenic bacteria can be transferred to other bacteria through plasmids or phages, helping pathogens to cause infectious diseases (Wagner and Waldor, 2002; Chen et al., 2021; Kim et al., 2022). The metagenomics technique has advantages in exploring the presence of ARGs, MGEs, VFGs, and resistomes from various environments (Segata et al., 2012; Ma et al., 2014; Bashiardes et al., 2016; Sukumar et al., 2016). The investigation of ARGs and VFGs in such environments helps to understand the possible human health risks associated with genes that exist in these environments and have the ability to spread to other regions.

Furthermore, metagenomics studies have facilitated researchers to identify many beneficial microbes in river sediment for bioremediation properties, plastic-degrading microbes, and classes of ARGs (Iyer and Damania, 2020; Cabral et al., 2019; Behera et al., 2021; Das et al., 2020). However, there has been limited exploration of the metagenomics of microbial communities, their functions, and potential ARGs in the Brahmaputra River, India. Therefore, the present research aimed to investigate the bacterial communities in the Brahmaputra River and explore the relationship between bacterial composition and their functional potential across different stretches by screening functional genes associated with ARGs. The study also explored MGEs, mainly plasmid-related genes and virulence factor genes (VFGs), derived from the sediment metagenome.

## Materials and methods

### Study area and collection of samples

**Figure 1:**
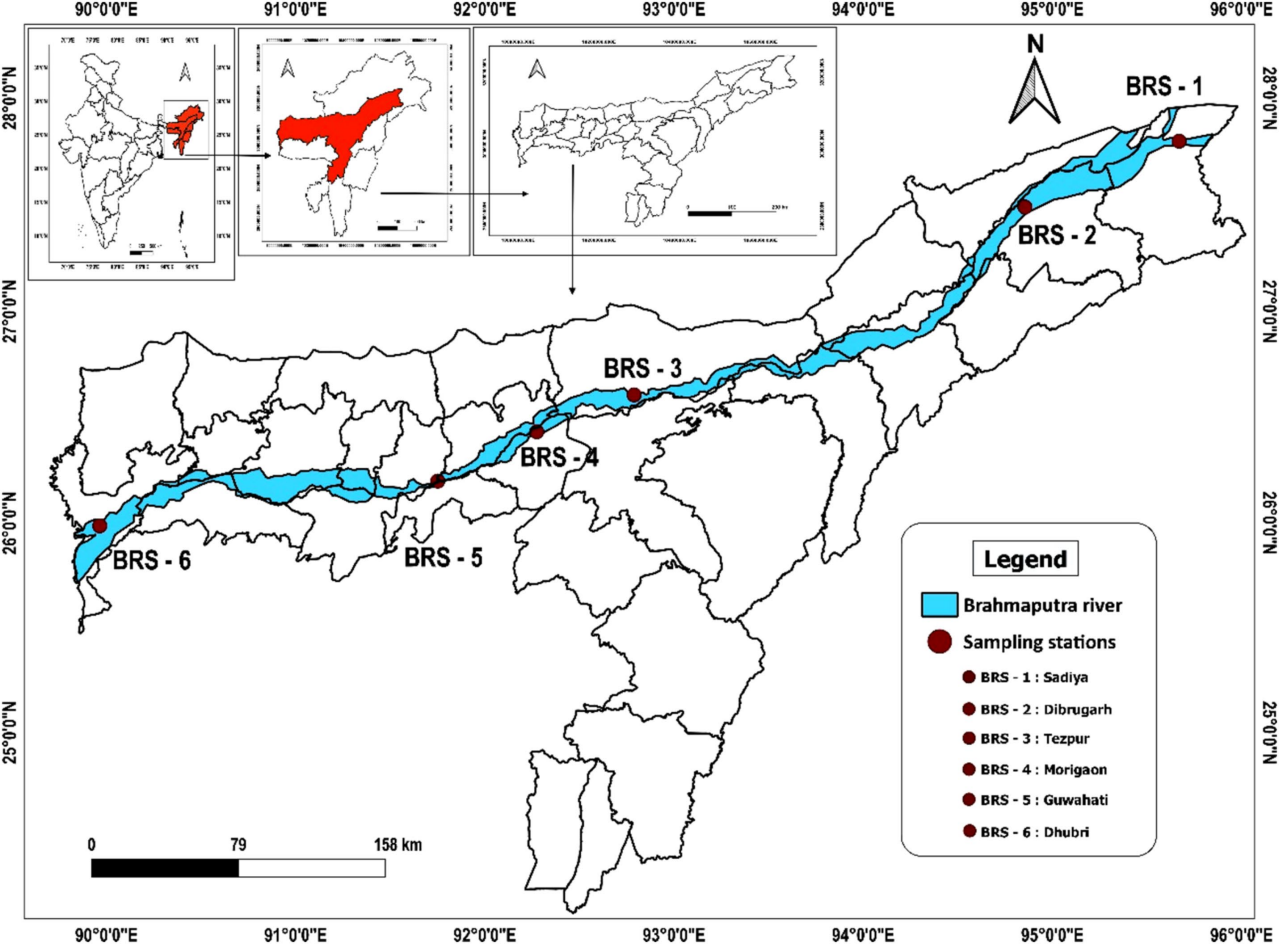
Map showing sampling sites of Brahmaputra River, India.

Sediment samples were collected from six locations of the Brahmaputra River, India, covering the major landing centers of its upper (Sadiya and Dibrugarh), middle (Tezpur and Morigaon), and lower (Guwahati and Dhubri) stretches between September and October 2021 (Figure 1). The sampling locations were as follows: Sadiya (Code: BRS-1; 27° 49.14” N 95° 40.52″ E), Dibrugarh (Code: BRS-2; 27° 29.9” N 94° 54.13″ E), Tezpur (Code: BRS-3; 26° 36.58” N 92° 47.27″ E), Morigaon (Code: BRS-4; 26° 17.20” N 92° 06.40″ E), Guwahati (Code: BRS-5; 26° 11.43” N 91° 45.20″ E), and Dhubri (Code: BRS-6; 26°1.20” N 89° 59.41″ E). At each sampling location, sediment samples were collected from five distinct points at a depth of 15–20 cm, resulting in a single composite sample of approximately 500 g. This approach aimed to accurately represent the microbial diversity present at each specific sampling location. The samples were transported in sterile containers within an icebox and stored at −80°C for further laboratory analysis.

### Physicochemical parameters analysis

Water samples were collected from all six sampling sites of the Brahmaputra River using clean 1,000 ml polypropylene bottles for the determination of various physicochemical parameters. The river water samples were analyzed for temperature (°C), dissolved oxygen (DO), pH, total dissolved solids (TDS), salinity (%), specific conductivity (μS/cm), biological oxygen demand (BOD), and chemical oxygen demand (COD) as per the standard methods of American Public Health Association (APHA) (APHA AWWA WEF, 2012). To collect sediment samples, an Ekman dredger was used. Three samples were collected from each sampling site. To obtain uniform composite samples, the residue was mixed thoroughly. The samples were transported in plastic pouch bags to the laboratory for further examination. The sediment samples were analyzed as per APHA standard methods for texture, pH, specific conductivity, organic carbon, available phosphate, and total nitrogen (APHA AWWA WEF, 2012). For heavy metal analysis, the sediment samples were preserved with concentrated HNO_3_ and analyzed using inductively coupled plasma mass spectrometry (ICP-MS) (NexION 1,000, PerkinElmer, Waltham, United States).

### Extraction of DNA and high-throughput sequencing

Total genomic DNA was isolated separately from six sediment samples using Power soil DNA isolation kits (Qiagen, Germany) as per the manufacturer’s protocol. The purity and integrity of isolated DNA were assessed using agarose gel electrophoresis (0.8%) and by absorbance (260/280 ratio) in a Nanodrop-2000 spectrophotometer (Thermo Scientific, Burladingen, Germany). Six DNA libraries, each representing a different sampling location, were prepared using the Truseq Nano library preparation kit (Illumina #20015964). The libraries were quantified using a Qubit fluorometer (Thermofisher, Life Technologies, CA, United States) and a DNA HS assay kit (Thermofisher #Q32851, United States), following the manufacturer’s protocol. The insert size of each library was estimated using Tapestation 4150 (Agilent, Waldbronn, Germany) (Agilent # 5067-5582) according to the manufacturer’s protocol. Subsequently, the libraries were indexed using barcodes and sequenced using the Illumina NovaSeq 6,000 platform to generate paired-end reads.

### Data submission

The raw metagenomic sequence data, generated in the study was submitted to the National Center for Biotechnology Information (NCBI) Sequence Read Archive (SRA) with BioProject identification number PRJNA944918. Each sediment metagenome BRS (1–6) can be assessed through SRA accession numbers (SAMN33764823, SAMN33764824, SAMN33764825, SAMN33764826, SAMN33764827, and SAMN33849149), respectively.

### Sequence analysis and taxonomical binning

The sequence reads underwent preprocessing to remove the adaptors, poor-quality bases (quality value [QV] < 20 Phred score), and short reads using the fastP tool (version 0.23.2) (Chen et al., 2018). Later, the high-quality reads were assembled using MEGAHIT version 1.2.9 (Li et al., 2015) for *de novo* metagenome assembly. The resulting assembled contigs were subjected to the taxonomic assignment using the Kraken2 tool (version 2.1.1) with background database prebuilt reference sequence (RefSeq) indices: PlusPF (version 2022.06.07). Kraken2 assigns the taxonomic labels to the contigs on their k-mer content, comparing them to the reference genome sequences. The Kraken-mpa-report tools (version 1.2.4) were utilized to summarize read counts across taxonomic ranks for multiple samples (Wood and Salzberg, 2014).

### Functional annotation

The bioinformatics database Kyoto Encyclopedia of Genes and Genomes (KEGG) was used to annotate and interpret functional genes and metabolic pathways in metagenomic data using best hit with a known reference sequence (Kanehisa et al., 2017). With over 13,000 nodes, the KEGG classification is represented as a rooted tree—the leaves of which stand for several roots. Clusters of Orthologous Groups (COG) uses orthology to categorize proteins into functional groups, which helps with metagenomic investigations’ functional annotation of genes. The SEED database facilitates the discovery of metabolic pathways and functional potential in metagenomic datasets by offering functional annotations and subsystem classifications for microbial genomes. The analysis has been carried out using MEGAN 6 (Huson et al., 2007).

### Detection of ARGs, MGEs-plasmid type, and VFGs from sediment metagenome of Brahmaputra River

The tool Abricate (version 1.0.1) (https://github.com/tseemann/abricate) was used to detect antibiotic resistance genes (ARGs), MGEs-plasmid type, and virulence factor genes (VFGs) in the metagenome by mass screening the assembled contigs against the Comprehensive Antibiotic Resistance Database (CARD) with minimum DNA percentage identity and percentage coverage of 80 and 70, respectively, for ARGs, PlasmidFinder (Carattoli et al., 2014) for MGEs-plasmid type and the virulence factor database (VFDB) for VFGs. For ARGs heatmap generation, the R ggplot2 package was used (Wickham, 2009). The input values for the heatmap were derived from antibiotic resistance genes (ARGs) identified using the ABRicate tool with the CARD. For each sample, we considered the top percentage identity value for each detected ARG. This means that the highest percentage identity match between the sample and the reference gene in the CARD database was used as the input for the heatmap. The color scale represents percentage identity values, ranging from green (lower identity) to red (higher identity), highlighting the strength of the match for each gene. This approach allows clear visualization of the strongest matches for each ARG in the different sample groups Brahmaputra River station 1 to station 6 based on their top percentage identity.

### Statistical analysis

The difference in bacterial composition between samples was calculated using principal component analysis (PCA). The diversity indices (Fisher, Simpson, Chao1, and Shannon) were used to calculate the *α*-diversity across all the samples. The variation in species between bacterial composition was calculated using *β*-diversity indices. The multivariate statistical tool canonical correspondence analysis (CCA) was used to quantify the relationship between the relative abundance of microbes with water and sediment parameters. The pattern of co-occurrence among ARG subtypes was investigated using a network inference approach based on strong correlations (*ρ* > 0.8) and high statistical significance (*p* < 0.01), following the methodology outlined by Junker and Schreiber (2008).

## Results

### Metagenomics sequencing and assembly

The number of reads varied from 31.28 million (BRS-2; an upper stretch of the river) to 80.53 million (BRS-4; a middle stretch of the river), with an average read of 54.98 million reads per sample. The maximum number of scaffolds was found in BRS-6, and the minimum number in BRS-2. The high-quality reads (30 > Q) were assembled into contigs, and a total of 0.37 million contigs were generated, including all samples. The minimum and maximum lengths of the contig are 200 and 123,389, respectively, with a mean value of 61,397 (Table 1).

**Table 1:** Summary of sequence assembly statistics.

Parameters	BRS-1	BRS-2	BRS-3	BRS-4	BRS-5	BRS-6
Number of reads	45,094,339	31,286,856	42,980,657	80,530,119	49,806,226	80,221,151
Number of contigs	314,441	153,185	327,294	209,498	213,305	416,350
Contig length (minimum)	200	200	200	206	200	200
Contig length (maximum)	71,416	39,539	37,234	35,363	123,389	61,444
N50	652	786	818	790	887	687
N90	520	531	533	532	537	524
Overall GC content (guanine-cytosine content)	59.62	61	63.71	62.98	60.2	61.02
Genome fraction (%)	13.391	39.504	13.028	20.952	26.544	7.837

### Microbial community structure, diversity, and richness

**Figure 2:**
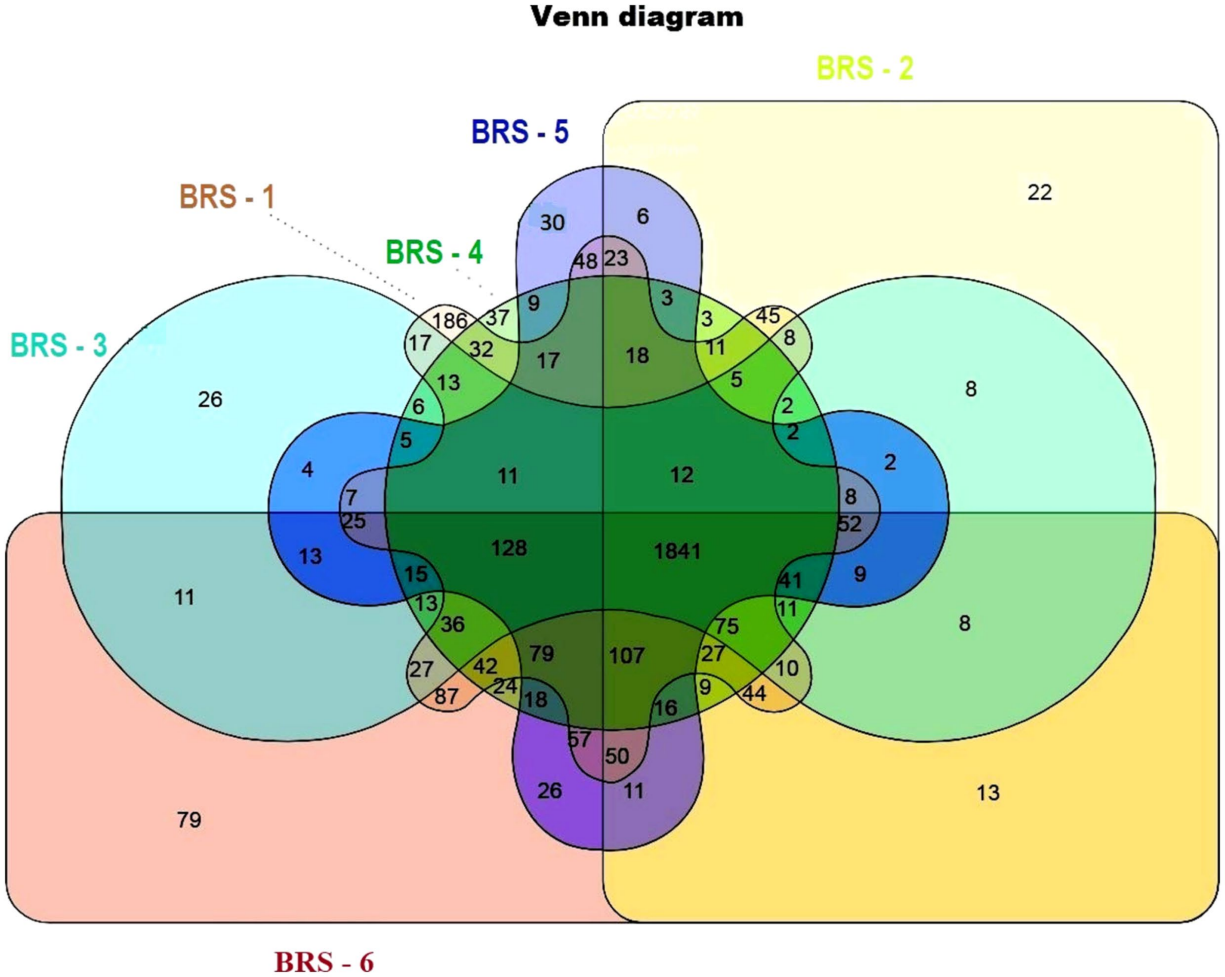
Venn diagram showing unique microbes in the six sampling sites. Different colors indicate different sampling sites; light brown color indicates BRS-1; light green color indicates BRS-2; light blue indicates BRS-3; dark green indicates BRS-4; dark blue indicates BRS-5; and dark red indicates BRS-6. A combination of colors indicates the union of two or more sites.

**Figure 3:**
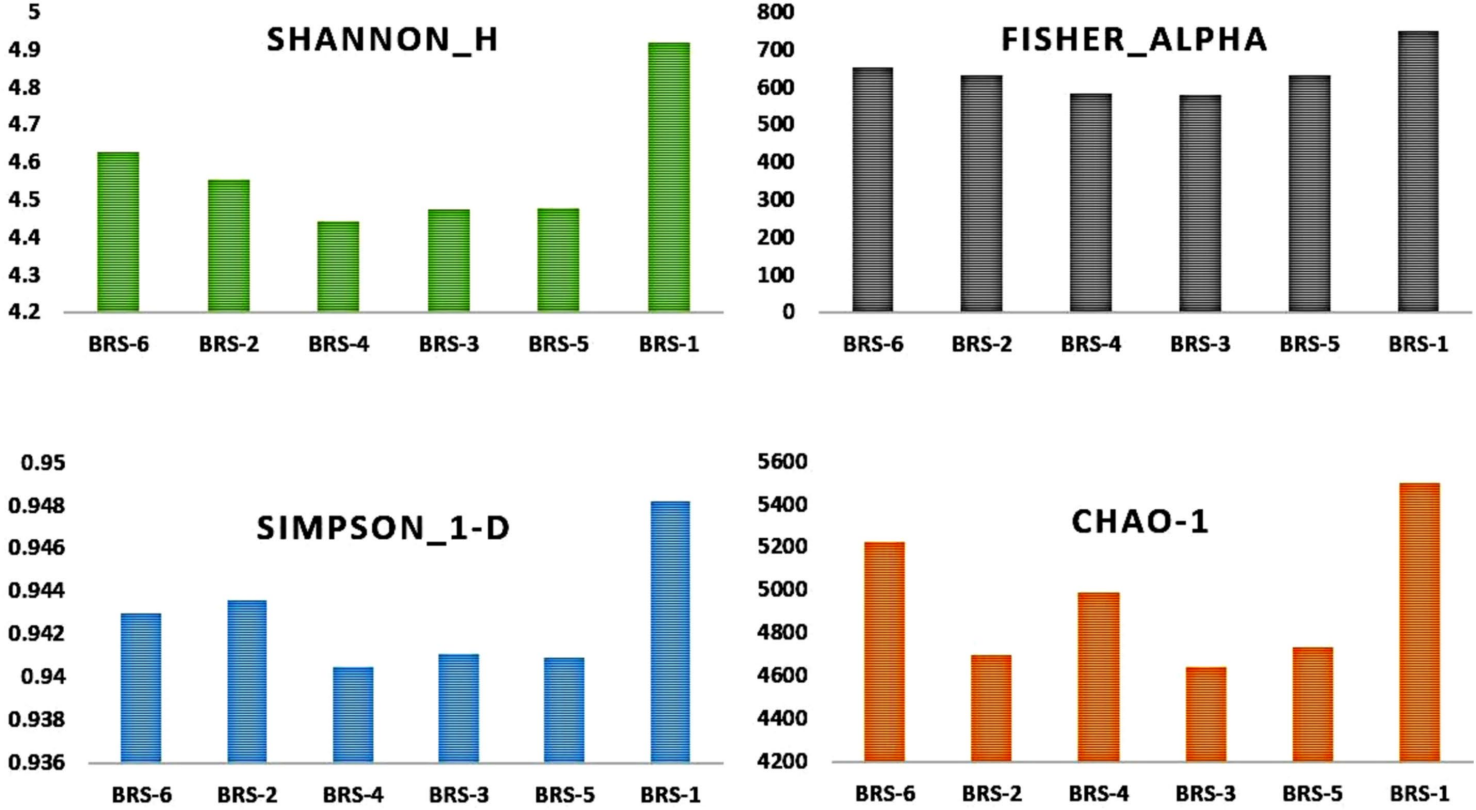
*α*-Diversity of sediment metagenome of Brahmaputra River, India. α-Diversity indices: parametric indices (Shannon and Simpson) and non-parametric indices (Fisher and Chao1) are shown in the bar diagram.

**Figure 4:**
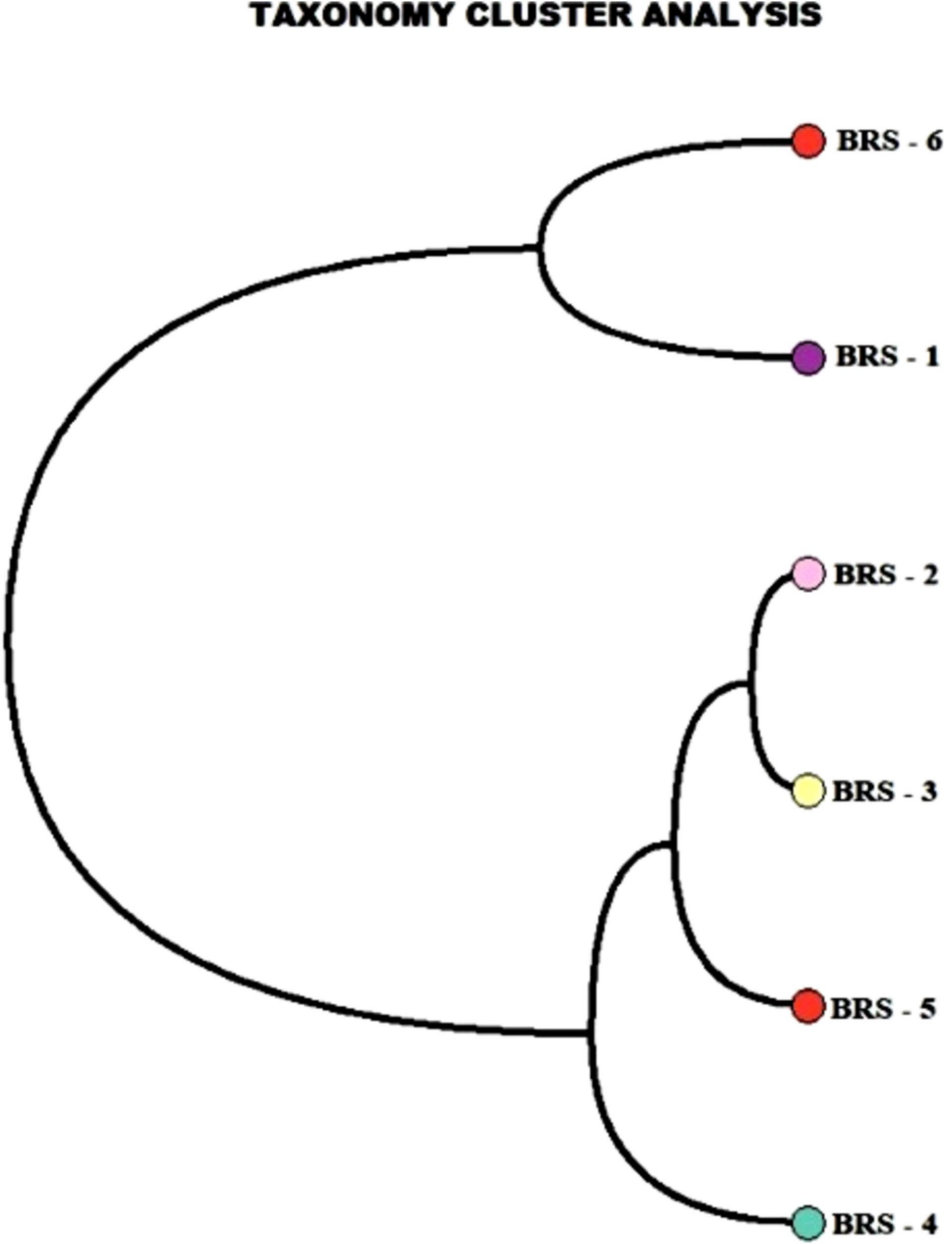
Taxonomic clustering of microbial communities in different sampling sites.

The number of taxon abundance values varied from 2,451(BRS-3) to 3,148 (BRS-1) with a mean value of 2,744. Of these, 1,841 taxon abundance values were found to be common among all the samples. A large number of unique taxon abundance values were observed in the sample BRS-1 (186 numbers), followed by BRS-6 (79 numbers), BRS-3 (37 numbers), BRS-5 (30 numbers), BRS-4 (26 numbers), and BRS-2 (22 numbers), respectively (Figure 2). Accordingly, the Shannon’s H index was relatively high in BRS-1 (upper stretch) followed by BRS-6 (lower stretch of the river). The non-parametric diversity indices, that is, Fisher’s *α* and Chao1, were found maximum in the origin of the upper stretch of the river (BRS-1) and lowest in the middle stretch (BRS-3) (Table 2; Figure 3). Taxonomic cluster analysis showed the similarities in microbial communities among the sampling sites. BRS-1 and BRS-6 clusters under the same clade show similar groups of microbes than the other clade formed by BRS-2, BRS-3, BRS-4, and BRS-5 (Figure 4).

**Table 2:** Summary of microbial diversity indices.

Parameter	BRS-1	BRS-2	BRS-3	BRS-4	BRS-5	BRS-6
Taxon abundance values	3,148	2,502	2,451	2,668	2,693	3,004
Simpson’s Index	0.9482	0.9436	0.9411	0.9405	0.9409	0.943
Shannon’s Index	4.628	4.556	4.476	4.442	4.48	4.628
Chao1	5,497	4,698	4,644	4,987	4,733	5,224
Fisher’s α-diversity index	752.1	631.9	582.1	584.7	634	653.7

### Comparison of microbial community across the samples

**Figure 5:**
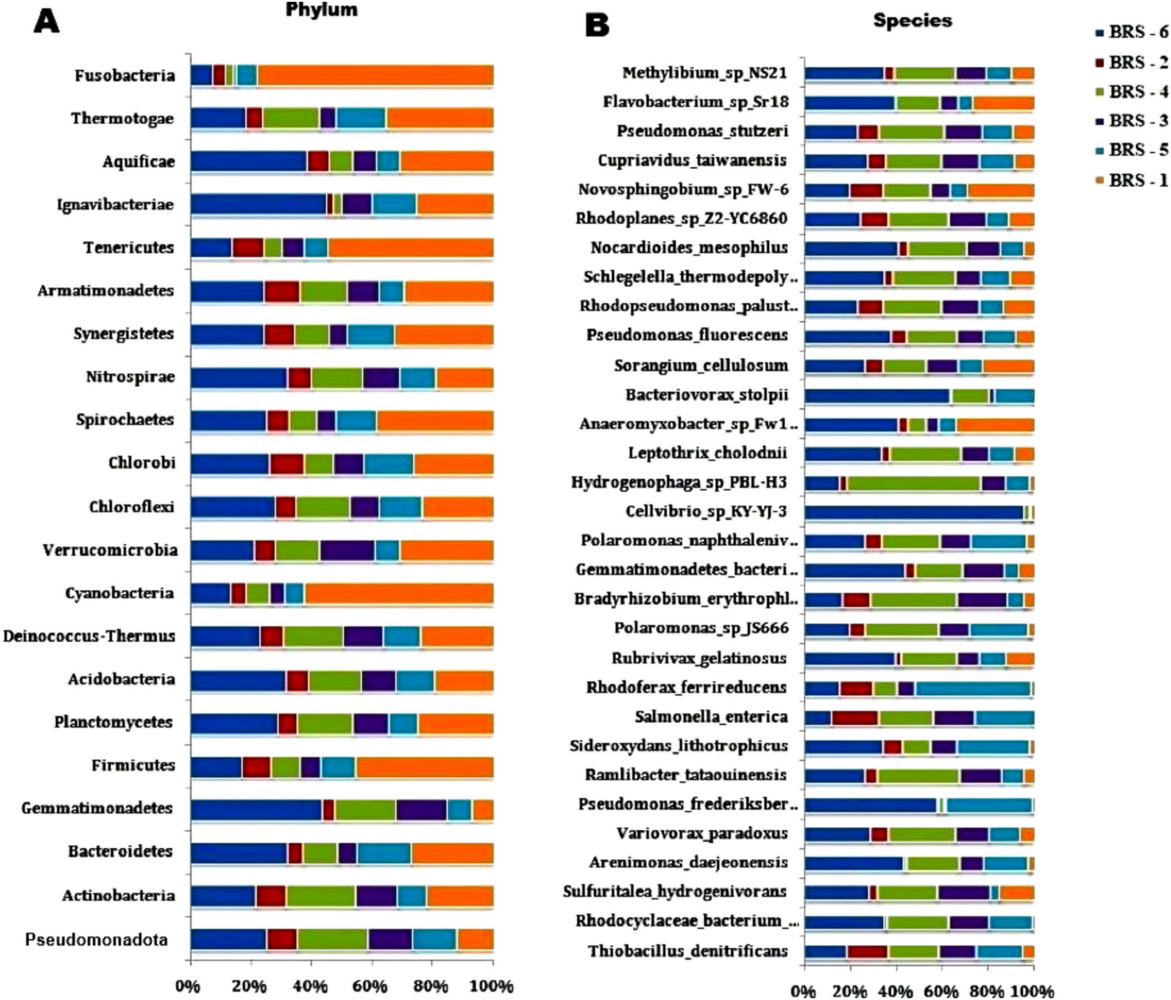
The figure shows two stacked bar charts depicting the relative abundances of bacterial taxa at the phylum and species levels across different sample groups (labeled BRS-1 to BRS-6). In both charts, the percentage scale along the *x*-axis shows the relative abundance (%) of each taxon per sample.

Among the microbial communities, the bacterial population dominates, contributing 99.22%, followed by archaea (0.40%), eukaryota (0.35%), and viruses (0.02%), respectively. A total of 31 phyla of bacteria dominated by Pseudomonadota, Actinobacteria, Bacteroidetes, Firmicutes, Cyanobacteria, and Planctomycetes were observed in all the samples. The relative abundance of the phyla varied from 62.47 to 83.48% (Pseudomonadota), 11.10 to 24.89% (Actinobacteria), 0.97 to 3.82% (Bacteroidetes), 0.54 to 3.94% (Firmicutes), 0.14 to 1.70% (Cyanobacteria), and 0.30 to 0.78% (Planctomycetes) across the samples (Figure 3). In Archaea, Euryarchaeota (from 91.68 to 97.05%) dominates, followed by Thaumarchaeota (from 0.59 to 5.91%) and Crenarchaeota (from 2.36 to 5.05%), respectively (Figure 5A). At the class level, the five bacterial classes *β*-proteobacteria, Gammaproteobacteria, α-proteobacteria, Actinobacteria, and Deltaproteobacteria are the most dominating categories in all the samples. In addition, in the Archaean class, Methanomicrobia and Halobacteria dominate among all the samples.

At the genus level, significant variations in the bacterial composition across all sampling sites are observed. Some of the prominent genera are *Pseudomonas*, *Salmonella*, *Bradyrhizobium*, Var*iovorax*, *Hydrogenophaga*, *Burkholderia*, *Cupriavidus*, *Thiobacillus*, *Mycobacterium*, and *Streptomyces* in all the sampling sites. While the majority of the dominant species are unknown (uncultured), *Thiobacillus denitrificans* is the most abundant in all the sites, *Salmonella enterica* dominates in all sites except in BRS-1. *Rhodocyclaceae bacterium*_*PG1-Ca6* dominates in all except in BRS-1 and BRS-2; *Pseudomonas frederiksbergensis* and *Arenimonas daejeonensis* dominate in BRS-6 site; *Bradyrhizobium erythrophlei* dominates in BRS-2, BRS-3, and BRS-4; *Ramlibacter tataouinensis* dominates in BRS-3, BRS-4, and BRS-6; *Polaromonas_sp_JS666* dominates in BRS-3, BRS-5, and BRS-6; *Variovorax paradoxus* dominates in BRS-4 and BRS-6 and *Novosphingobium sp_FW-6* dominate only in BRS-1. The relative abundance of the most abundant bacterial species is shown in Figure 5B.

**Figure 6:**
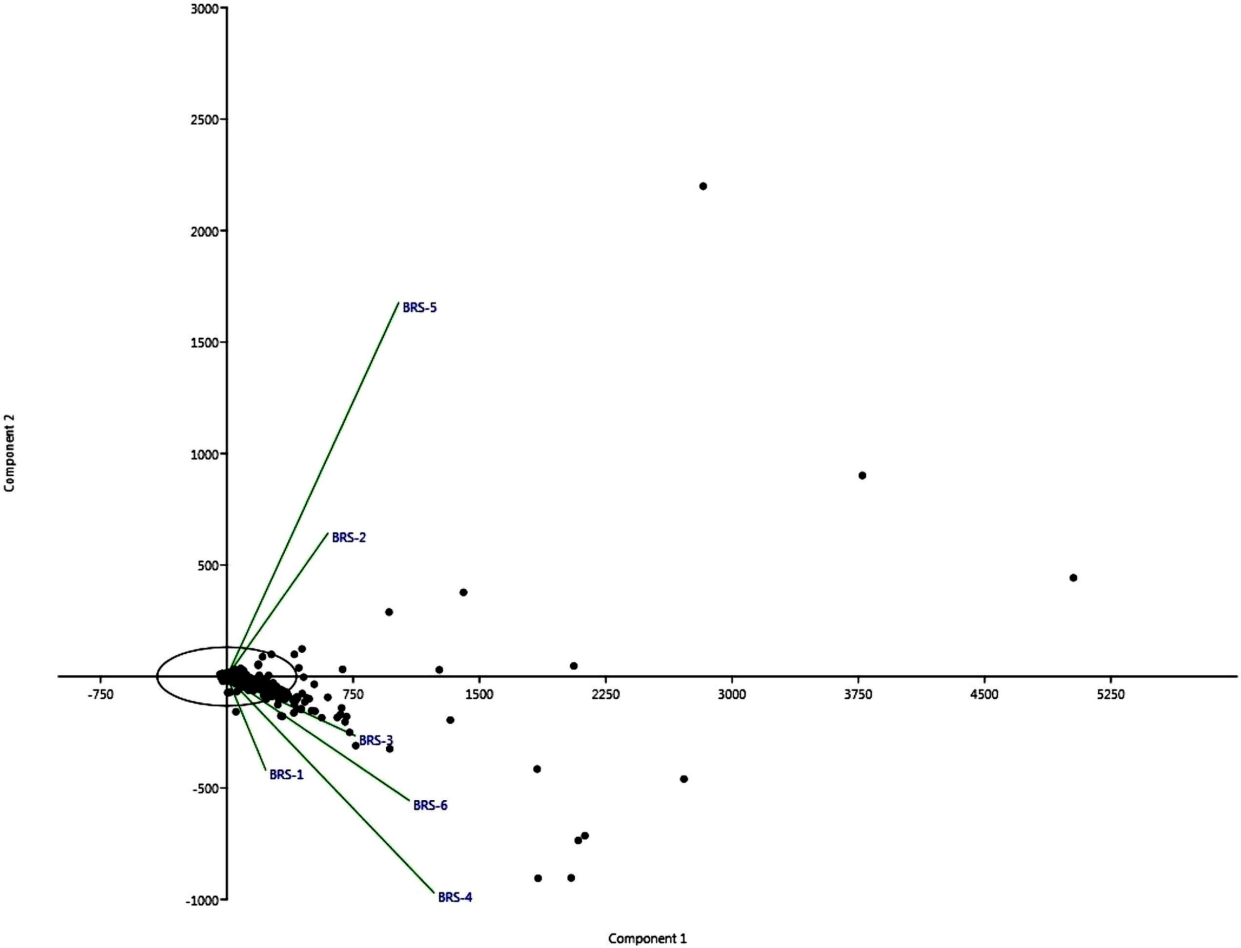
The figure shows two stacked bar charts depicting the relative abundances of bacterial taxa at the phylum **(A)** and species **(B)** levels across different sample groups (labeled BRS-1 to BRS-6). In both charts, the percentage scale along the x-axis shows the relative abundance (%) of each taxon per sample.

The difference in bacterial composition between samples was calculated using PCA. The findings highlighted significant differences between river sampling sites. The proportion of the total variability among the samples accounted for PC1 and PC2 is 90.79%. Two sediment samples obtained from BRS-2 and BRS-5 sites, which are the populated sites of the River, were positively correlated based on the bacterial community to component 2. However, the samples from the other four sites, BRS-1, BRS-3, BRS-4, and BRS-6, were clustered together and negatively correlated with the component 1 (Figure 6).

### Correlation of microbial community and physicochemical variables

We used CCA to determine the relative abundance of the microbial community at the phylum level based on values of water and sediment parameters measured at the site, which allows us to identify quantifiable relationships between microbial communities and physiochemical parameters of sediment and water quality. The water and soil quality parameters were summarized in Supplementary Tables S1, S2. Physicochemical parameters were found to be acceptable for the survival and growth of aquatic organisms. At the phylum level, Verrucomicrobia, Deinococcus-Thermus, Crenarchaeota, Armatimonadetes, and Planctomycetes formed a major group and associated more with water parameters, such as dissolved oxygen, pH, water temperature, free CO_2_, turbidity, TDS, nitrate and nitrite and Gemmatimonadetes are shown to be associated with phosphate (Supplementary Figure S1). In sediment, the majority of the parameters, namely, available nitrogen, available phosphate, free CaCO_3_, heavy metals, pH, and organic carbon, are associated with microbial groups Deinococcus-Thermus, Crenarchaeota, Pseudomonadota, Nitrospirae, and Acidobacteria, whereas Verrucomicrobia is associated with Arsenic (Supplementary Figure S2).

### Functional profiling of microbes

**Figure 7:**
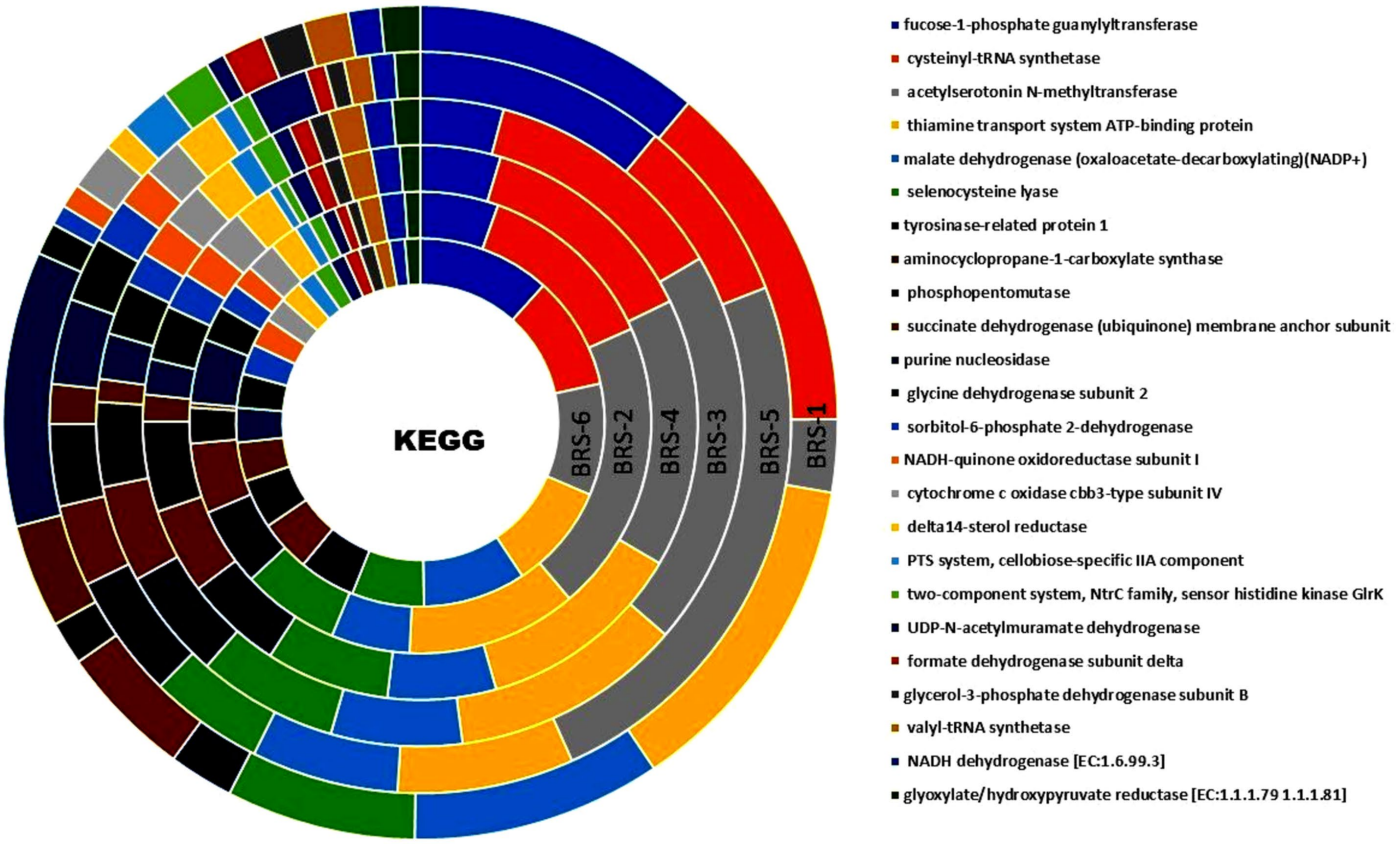
Functional genes from metagenome data enriched using KO terms from the stretches of the Brahmaputra River through KEGG orthologous pathways.

Functional annotation of sequences of all six metagenome samples through KEGG databases revealed several important functional features. A total of 407,034 (BRS-1), 291,345 (BRS-2), 414,113 (BRS-3), 673,709 (BRS-4), 410,221 (BRS-5), and 722,445 (BRS-6) numbers of genes were assigned to KEGG pathway. A total of eight major metabolic pathways were identified using KEGG analysis. Based on KEGG annotation, the relative abundance of the top six metabolic pathways were metabolism (from 44.02 to 33.85%), environmental information processing (from 2.73 to 1.62%), genetic information processing (1.30 to 0.73%), human diseases (0.59 to 0.20%), cellular processing (0.44 to 0.10%) and organismal systems (0.48 to 0.42%). The highest level of KEGG analysis showed that the major metabolic pathways involved are alcohol dehydrogenase (NADP+), malate dehydrogenase (oxaloacetate-decarboxylating) (NADP+), succinate dehydrogenase (ubiquinone) membrane anchor subunit pyrroline-5-carboxylate reductase, nitrate reductase gamma subunit, fucose-1-phosphate guanylyltransferase and cysteinyl-tRNA synthetase are highly prominent across all sampling sites highlighting their role in energy and amino acid metabolism, central carbon metabolism, stress response pathways and degradation (Figure 7). Overall, these findings provide a thorough overview of important biochemical regulatory networks that are active at the study sites, reflecting the organism’s metabolic diversity and environmental adaptability (Figure 7).

**Figure 8:**
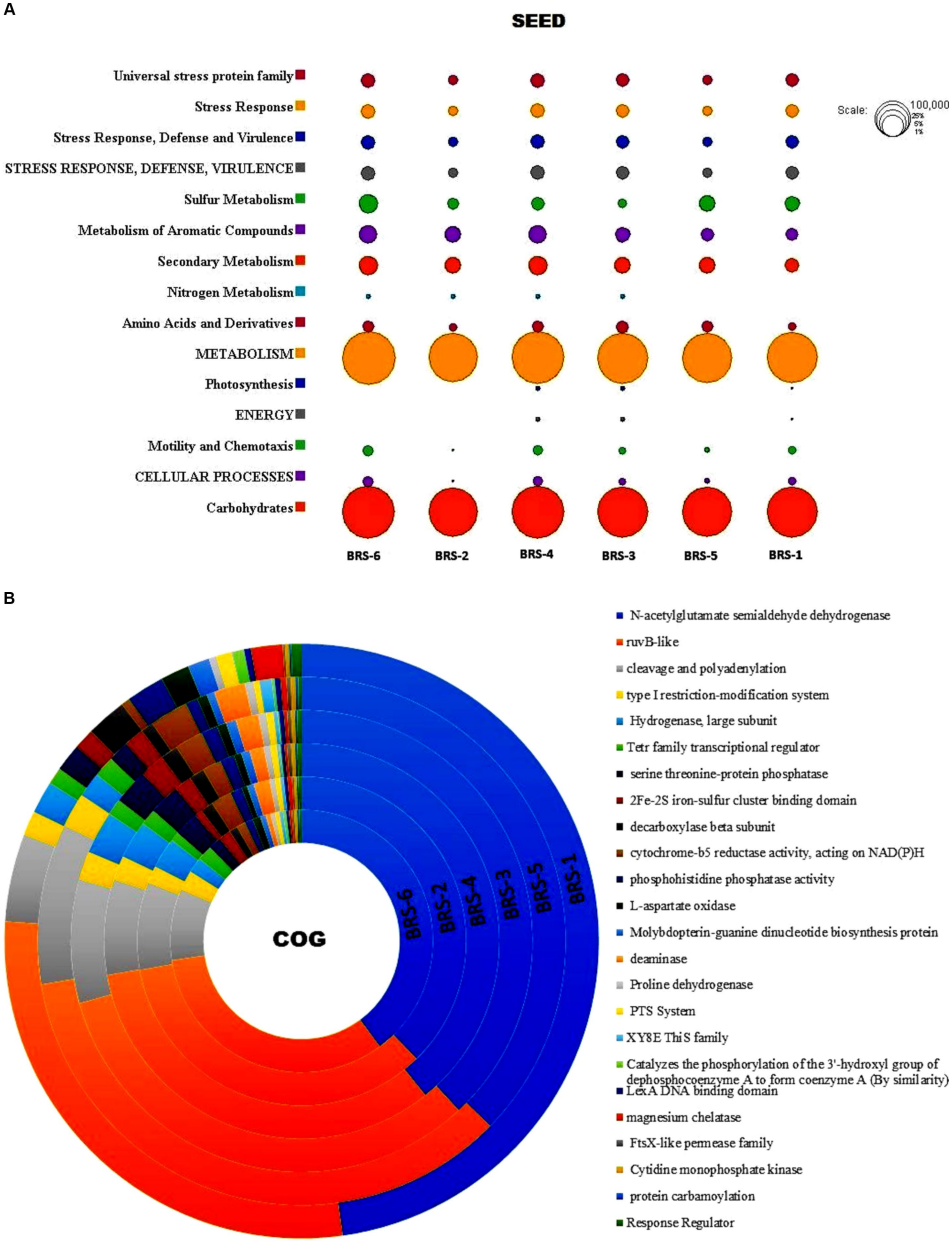
**(A)** Functional classification of microbes from Brahmaputra River through SEED tool. Circle size is based on the number of assigned values for particular functions; **(B)** Functional classification of microbes from Brahmaputra River through COG subsystem.

Functional classification through SEED revealed 89,204 (BRS-1), 57,373 (BRS-2), 82,793 (BRS-3), 134,077 (BRS-4), 81,239 (BRS-5), and 147,565 (BRS-6) genes were assigned to six sediment metagenome of Brahmaputra River. SEED classification also revealed the presence of many functional genes in metagenome data from river sediments (Figure 8A). Among them, genes associated with metabolism, carbohydrates, secondary metabolism, stress response, defense, virulence, energy, and cellular process were found to be more significant than the other functions in all the sampling sites. Some of the functions were found to be less significant in a few of the sites; for example, nitrogen metabolism is found to be less in sites BRS-5 and BRS-1, and energy function is also found to be less significant in BRS-2, BRS-5, and BRS-6. COG categories assigned 2,69,246, 1,95,054, 275,570, 434,904, 2,65,255, and 4,67,374 number of genes in the metagenome of river sediment. A total of 10,57,976 (63.83 to 53.91%) genes are related to metabolism in all the samples, while 6,90,906 (from 41.59 to 27.06%) genes are related to information storage and processing, and 1,58,521 (from 9.09 to 7.24%) are associated with cellular processing and signaling. The deeper level of COG analysis revealed that several pathways, including N-acetylglutamate semialdehyde dehydrogenase, RuvB-like proteins, cleavage and polyadenylation, and the type-I restriction-modification system, were prominently represented across all sampling sites indicating diverse functional capabilities and adaptive strategies of the microbial communities. A comprehensive overview of the other major pathways can be found in Figure 8B.

### Mining of antibiotic resistance genes, plasmid-related genes, and virulence factor genes

**Figure 9:**
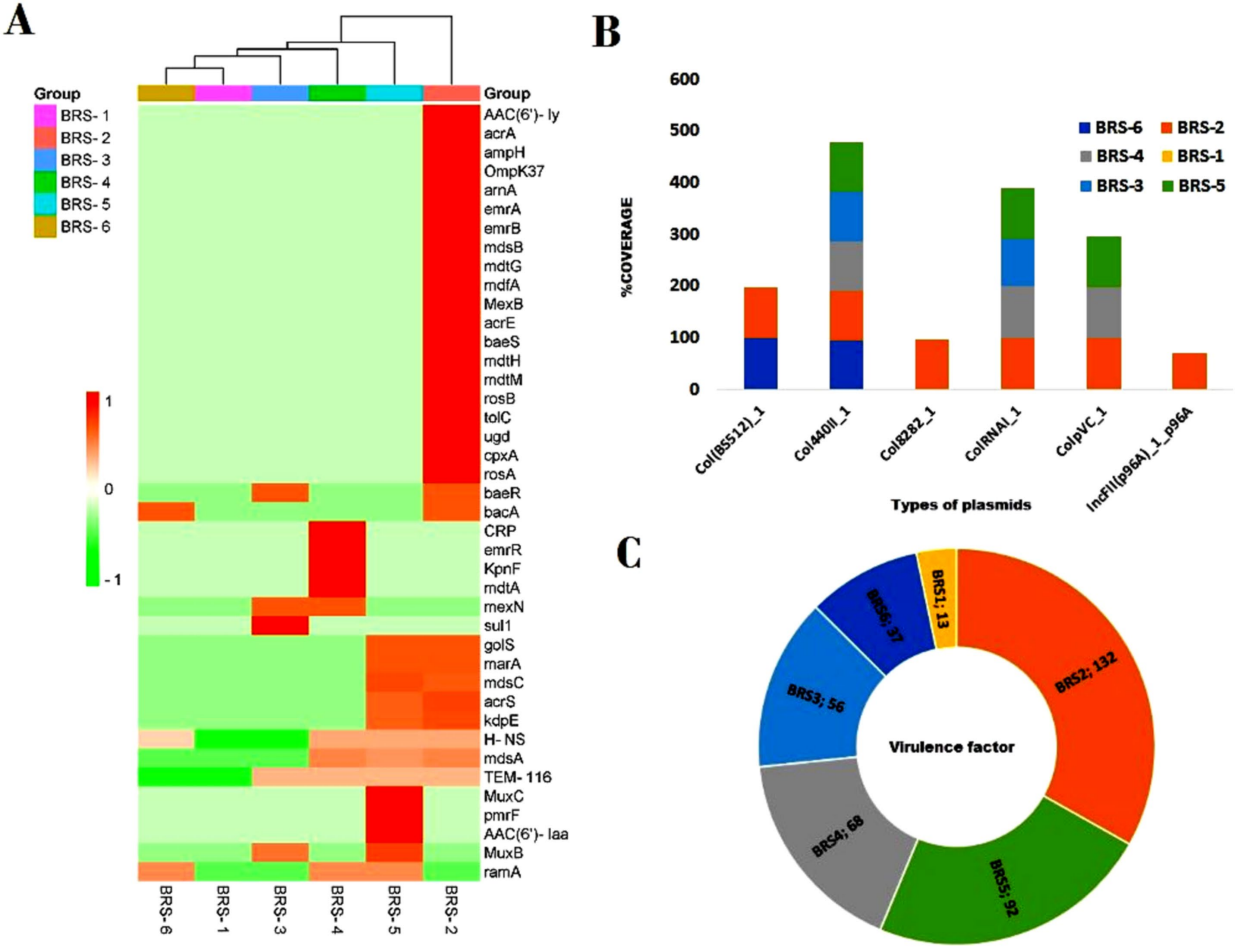
Abundance of ARGs, plasmid types, and virulence factors in all the six selected sites of Brahmaputra River, India. **(A)** The heatmap shows variability in antibiotic resistance genes (ARGs) profiles across samples, with some samples showing more pronounced resistance patterns. The color scale on the left indicates the presence of ARGs based on percentage identity, ranging from green (lower identity) to red (higher identity). The *y*-axis lists the resistance genes. The *x*-axis represents the sample groups (BRS-1 to BRS-6), with a dendrogram at the top, indicating clustering based on similarity in resistance gene profiles. The dark red coloring indicates samples showing a high abundance of specific genes are indicated by; **(B)** The diversity in plasmid types, with different plasmids dominating in different samples, indicating potential plasmid-mediated gene transfer; **(C)** The distribution of virulence factors (VFs), with some samples (BRS-2 and BRS-5) having significantly higher counts, suggesting a greater virulence potential.

A total of 50 diverse antibiotic resistance genes (ARGs) were identified, including all sampling locations. These genes include multidrug resistance genes (MDR), aminoglycoside resistance (AGR), *β*-lactam resistance (BLR), fluoroquinolone resistance (FR), phenicol resistance (PR), rifampicin resistance (RR), sulfonamide resistance (SR) and tetracycline resistance (TER) genes generally and ARGs, namely, *AAC (6′)-ly*, *CRP*, *acrA*, *H-NS*, *TEM-116*, *emrB*, *emrR*, *golS* and *mdsA*, *mdsB*. A list shows genes linked to significant antibiotic groups (Table 3). The heatmap shows the presence/absence of various antibiotic resistance genes across the six sample groups (BRS-1 to BRS-6) based on their percentage identity (Figure 9A). These sampling sites pass through the major city areas, whereas the start point (BRS-1) and endpoint (BRS-6) sampling sites contain very few ARGs. Approximately 44% of the ARGs were resistant to two or more drug classes. The gene *TEM-116* was dominant in all the sites except in BRS-1. The genes *acrA*, *AAC (6′)-ly*, *ampH*, *H-NS*, *emrB*, *golS*, *mdsA*, and *mdsB* were predominant in BRS-2. The abundant ARGs at site BRS-4 were *CRP*, *ramA*, *mexN*, *H-NS*, *emrR*, and *mdsA*. The sampling site BRS-5 harbored more *mdsC*, *golS*, and *H-NS*. Genes such as *arr-1*, *arr-4*, and *vatB* were prevalent at BRS-1. The genes *bacA*, *ramA* were predominant in BRS-6 and genes *sul1* and *baeR* were predominant in BRS-3. Supplementary Table S3 is provided regarding the information on the detection of potential ARGs (including contig name, start, end of hit, fasta sequences, similarity percentage, coverage percentage, etc.). Out of the different resistance microbes, six microbial species, namely, *Escherichia coli*, *Enterobacter cloacae*, *Klebsiella pneumonia*, *Pseudomonas aeruginosa*, *Staphylococcus aureus*, and *S. enterica* showed resistance against multiple drugs. The highest resistance microbes abundances were recorded for *E. coli*, followed by *S. enterica* > *P. aeruginosa* > *K. pneumonia* > *Y. enterocolitica* > *S. aureus > V fluvialis* > *Mycolicibacterium smegmatis* and *E. cloacae*. Using the plasmid finder part of Abricate, we have identified fewer plasmid type-MGEs. The plasmid-related genes observed were Col (BS512), Col440II, Col8282, ColRNAI, ColpVC, and IncFII, respectively (Figure 9B). The plasmid-related genes were found to be highest in BRS-2 and negligible in BRS-1 sampling sites. The virulence factor analysis showed the presence of a total of 185 virulence factor genes (VFGs) mostly present in the BRS-2 sampling site (135 numbers), followed by BRS-5 (92 numbers), BRS-4 (68 numbers), BRS-3 (56 numbers), BRS-6 (37 numbers) and BRS-1 (13 numbers), respectively (Figure 9C). A few of the VFGs present in all sampling sites were *acpXl*, *algU*, *cheB*, *cheY*, *entB*, *flgC*, *flgG*, *fliA*, *pilG*, and *pilH*, respectively. Detailed information about all the VFGs was provided in Supplementary Table S4.

**Table 3:** Major antibiotic groups, along with the potential resistance genes.

Antibiotic group	Potential-resistant genes
Aminocoumarin	*cpxA, baeR, tolC, mdtA, MexB, baeS, MuxB, MuxC*
Aminoglycoside	*cpxA, baeR, tolC, KpnF, kdpE, baeS, AAC(6′)-Iy, AAC(6′)-Iaa, aadA2*
Carbapenem	*MexB, ramA, marA, tolC, OmpK37, golS, mdsA, mdsC, mdsB*
Cephalosporin	*tolC, KpnF, acrE, MexB, sdiA, ramA, acrA, marA, KpnF, ampH, OmpK37, golS, mdsA, mdsC, mdsB, acrS, acrA, Tem-116, H-NS, mexY*
Penam	*CRP, tolC, acrE, MexB, sdiA, ramA, mdsA, mdsC, mdsB, acrA, H-NS, marA, OmpK37, MexB, golS, acrS, Tem-116, ampC1, H-NS, mexY*
Fluoroquinolone	*CRP, tolC, mdtH, acrE, sdiA, ramA, acrA, H-NS, MexB, marA, emrA, emrB, emrR, mdtK, mdtM, H-NS, mexY*
Monobactam	*mdsC, mdsB, mdsA, golS, OmpK37, marA, Tem-116, MexB, MuxB, ramA, MuxC*
Macrolide	*CRP, tolC, KpnF, MexB, MuxB, mexY, MuxC*
Rifamycin	*arr-1, arr-4, acrS, acrA, sdiA, ramA*
Tetracycline	*acrA, acrS, sdiA, mdfA, H-NS, MuxB, mexY, ramA, MuxC, mdfA*
Sulfonamide	*sul1*
Streptogramin	*vatB*

### Co-occurrence pattern between ARG subtypes

**Figure 10:**
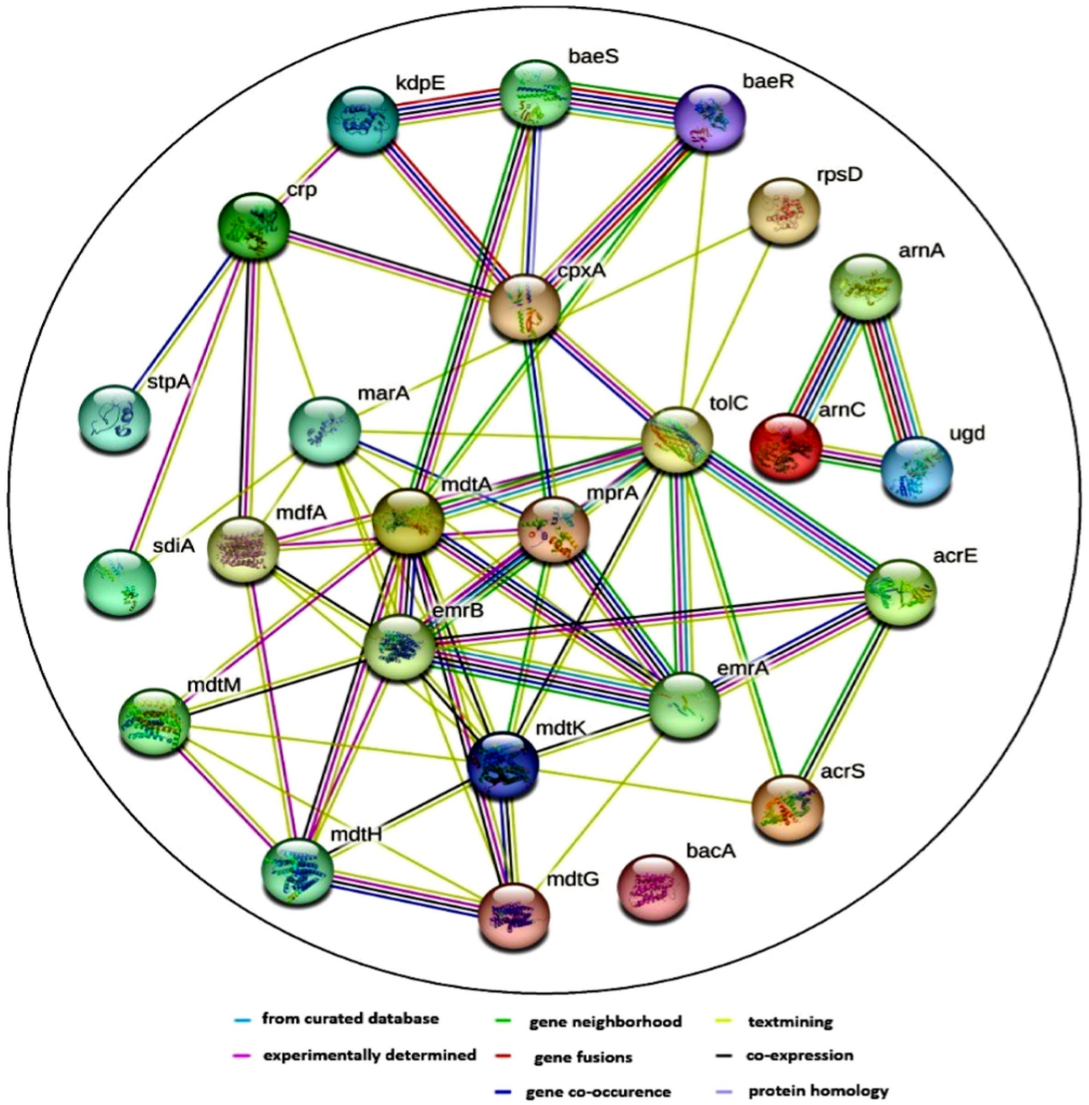
Protein–protein interaction (PPI) network of various antibiotic resistance genes (ARGs) and their co-occurrence patterns. The network was generated using the Search Tool for the Retrieval of Interacting Genes/Proteins (STRING) database, which predicts functional associations based on evidence from different sources. The nodes represent different microbial genes that are associated with antibiotic resistance mechanisms. The edges indicate different types of interactions. The color coding for the lines provides information about the nature of these interactions: Known interactions (blue) or experimentally determined (pink). Predicted interactions such as gene neighborhood (green), gene fusions (red), and gene co-occurrence (blue). Other interactions include text mining, co-expression, and protein homology (yellow, black, and cyan).

The pattern of co-occurrence among ARG subtypes was investigated using a network inference approach. The resulting network, depicted in Figure 10, comprises 25 nodes representing distinct ARG subtypes, connected by 66 edges indicating correlations. The average node degree, a measure of connectedness, is calculated at 5.28, suggesting that each ARG subtype is typically associated with around 5 other subtypes. Additionally, the average local clustering coefficient, indicating the prevalence of interconnected clusters, is found to be 0.653, signifying notable clustering within the network.

It was observed that ARG subtypes belonging to the same ARG type exhibit a tendency to co-occur. This phenomenon is exemplified by the correlation between *emrA*, *emrB*, *mdtA*, *mdtG*, *mdtH*, and *mdtK*, all associated with multidrug resistance. Furthermore, specific functional associations between ARGs become apparent; for instance, *arnA*, *arnC*, and *ugd*, conferring resistance to polymixin, are found to co-occur. Noteworthy interactions also involve *baeS* and *baeR*, which respond to envelope stress and activate various operons, including mdtABCD and potentially the CRISPR-Cas casABCDE-ygbT-ygbF operon. The ARGs’ subtypes revealed that the majority of them acted through multiple modes, such as antibiotic efflux, antibiotic inactivation, antibiotic target alteration, and replacement.

## Discussion

Microbial communities play an important role in ecological processes and maintenance of the diverse environments of river ecosystems. The river system’s continuous anthropogenic activity may have an effect on the composition and dynamics of the microbiome as well as their biological roles. The present study evaluated the microbial diversity and its functional profiling in sediments of the Brahmaputra River using shotgun metagenomic sequencing. The study also identified ARGs in the river sediments. The sediments from the Brahmaputra River sampling sites revealed greater diversity and abundance of microbial communities. The Shannon diversity indices were higher than the average value of 4, showing greater diversity. Similarly, Rout et al. (2022) found a higher diversity Shannon index value of up to 4 for microbial communities in the sediment of River Ganga, and Feng et al. (2022) also observed a greater Shannon index of the Bahe River Basin, China, than the average value of 6, indicating that the microbial community diversity in the sediments of the Bahe River Basin to be high. Bacterial populations are the most dominating among microbial communities in the sediments of the Brahmaputra River, contributing about 99%. Similarly, Behera et al. (2021) observed the occurrence of bacteria to be higher than other groups of microbes in River Ganga sediment. Using paired-end Illumina HiSeq, Malakar et al. (2021) sequenced the V3–V4 region of the 16S rRNA gene amplicon, identifying 631 genera of bacteria from 22 phyla found in the gills of Brahmaputra River fish, including both pathogenic and non-harmful species. Taxonomic profiling of bacteria in the study revealed the presence of phyla Pseudomonadota, Actinobacteria, Bacteroidetes, Firmicutes, Cyanobacteria, and Planctomycetes. The microbial taxonomic profile obtained in this study was consistent with other studies of microbial distribution in freshwater rivers, where bacterial population dominates the river sediment (Wang et al., 2017; Abia et al., 2018; Behera et al., 2021; Rout et al., 2022).

The most prevalent phyla across all sampling sites were Pseudomonadota and Actinobacteria, which is consistent with findings from several previous studies on river ecosystems (Reddy et al., 2019; Behera et al., 2020a,b; Feng et al., 2022). Pseudomonadota is commonly the abundant phylum in sediments, having a wide range of functions in nitrogen, carbon, and sulfur metabolism and degradation of organic and inorganic compounds (Spain et al., 2009; Bai et al., 2012; Zainun and Simarani, 2018). Among the Pseudomonadota classes, *β*-proteobacteria, Gammaproteobacteria, *α*-proteobacteria, and Deltaproteobacteria are the most dominating categories in all the sampling sites. A higher abundance of these classes of bacteria was also reported from lakes and rivers (Medeiros et al., 2016; Tsagaraki et al., 2018; Samson et al., 2019; Parida et al., 2022). *α*-Proteobacteria and β-proteobacteria abundance have been related to pH and nutrients of the aquatic environment (Newton et al., 2011). The nitrogen and sulfur cycles are significantly influenced by deltaproteobacteria and gammaproteobacteria (Mori et al., 2019; Masuda et al., 2022). The high abundance of gammaproteobacteria and deltaproteobacteria may also be linked to anthropogenic activities, primarily from domestic and industrial waste (Parida et al., 2022). Freshwater actinobacteria plays an important ecological role in degrading complex polymers, producing bioactive molecules, and recycling compounds (Trujillo et al., 2015; Lewin et al., 2016; Zothanpuia et al., 2018; Mawang et al., 2021). Additionally, Bacteroidetes and Firmicutes play a significant part in organic decomposition and fermentation (Oni et al., 2015; Yi et al., 2021). In Archaean phyla, Euryarchaeota, a common group of Methanomicrobiales found in freshwater environments, are abundant and help in the anaerobic degradation of organic matter (Koizumi et al., 2003; Bomberg et al., 2008). Similar observations were reported by Parida et al., 2022 in the Yamuna River and by Rathour et al. (2020) in Pangong Lake.

The presence of genera *Bradyrhizobium*, *Pseudomonas*, and Var*iovorax*, in the sediments of the Brahmaputra River indicates their role in denitrification and organic matter degradation (Lalucat et al., 2006; Horel et al., 2015; Liao et al., 2013). *Burkholderia*, *Hydrogenophaga*, and *Cupriavidus* have a role in biodegrading many kinds of environmental chemicals (Lambo and Patel, 2007; Han et al., 2015; Morya et al., 2020). At the species level, *T. denitrificans* was found to be abundant in all the sites, which has the capability for oxidation of sulfur, ferrous iron, and iron sulfides (Beller, 2005; Beller et al., 2006). *T. denitrificans* plays a key role in the nitrate-dependent oxidation of iron sulfide minerals in natural freshwater systems (Haaijer et al., 2006). The presence of *Salmonella enterica*, a foodborne pathogen, in all the sites except in BRS-1 (start of the river), indicates anthropogenic activities or polluted sites of the river as the river passes through the urban areas. Other abundant species, *Rhodocyclaceae bacterium*_*PG1-Ca6 and P. frederiksbergensis* degrade polycyclic aromatic hydrocarbons (Singleton et al., 2015; Ruiz et al., 2021), *Polaromonas_sp_JS666* degrades cis-1,2-dichloroethene in subsurface and aerobic environments (Giddings et al., 2010).

The *α*-diversity assessment, such as Simpson index, Fisher’s α-index, Shannon index, and Chao1 index, did not differ significantly in all the samples, with its highest richness at the upstream and downstream side of the river, and least at midstream. The maximum number of taxa was also observed at the start of the upstream and end of downstream of the river, forming one cluster, and the river sites passing through the urban areas formed one cluster. The undisturbed water with rich nutrients flows into the river contributing to the enrichment of microbial communities. Furthermore, unique and shared microbes among the sites show that the upstream contributed higher microbial species to downstream and midstream. A similar observation was reported in the Danube River, where upstream contributes more microbial diversity due to the passive transportation of microbial communities in lotic water and large contact of small headwaters facilitates the contribution of allochthonous bacteria to the river community (Savio et al., 2015). Chen et al. (2018) observed α-diversity increased gradually from upstream to downstream than the midstream, as microbial dispersal or drift limitations are influenced mainly by spatial distance between the sites.

Exploiting the functional potential of the genes expressed by the microbes helps to gain insight into the metabolic contribution of microbes to the river ecosystem. The present study’s functional profiling through the KEGG database revealed that the majority of the microbes were involved in metabolism and environmental information processing, degradation, and disease pathways. Similar findings were reported in the Apies River, South Africa (Abia et al., 2018), where bacteria were involved in metabolic and environmental information pathways, and some were related to human diseases involving infectious diseases. According to Raiyani and Singh (2020) the metabolic capabilities of microbial communities in Arabian seawater revealed several functions such as metabolism, genetic information processing, cellular processes, and deeper analysis revealed amino acid, carbohydrate, nucleotide metabolism, etc. Srivastava and Verma (2023) also identified significant genes that play roles in metabolism, degradation, and certain human diseases in the Ganga River. Several studies on functional profiles of environmental bacteria have been linked to human diseases, as discussed by Sandifer et al. (2015). The transmission of infectious diseases and antimicrobial resistance spread across the environment via wind, agricultural and urban runoff, and biological agents such as humans and animals (Allen et al., 2010). Based on the SEED and COG analysis, the microbiomes of the Brahmaputra River exhibited considerable enrichment in major pathways, including metabolism, secondary metabolism, information storage, and processing, which can be correlated with the prevalence of Pseudomonadota, Actinobacter, and Bacteroidetes in the river and its lakes (Koo et al., 2017; Ahmad et al., 2021; Parida et al., 2022).

The development of antibiotic resistance bacteria in aquatic ecosystems is caused by the direct discharge of hospital and domestic sewage, agricultural runoffs, and wastewater into open water bodies without adequate pre-treatment (Chandy, 2008; Prado et al., 2018). The development of multidrug-resistant strains, which enable the bacteria to adapt and survive, is a major health concern (Marathe et al., 2013). In the present study, through heatmap analysis, 50 numbers of ARGs’ subtypes were observed, and the majority of microbial groups are associated with multidrug resistance, aminoglycoside resistance, *β*-lactam resistance, fluoroquinolone resistance, phenicol resistance, sulfonamide resistance, rifampicin resistance, and tetracycline resistance. Similar observations were also reported by Das et al. (2020) where a high amount of ARGs were obtained from the Yamuna River, India. ARGs families in sediments of an urban river (Chen et al., 2019) and the European river (Kneis et al., 2022) reported resistance to aminoglycosides, macrolides, sulfonamide, or tetracyclines, possibly indicating the spread of ARGs in river environment because of selective pressure resulting from antibiotic use. In this study, six groups of plasmid-type MGEs were identified in the river sediment metagenome. Nguyen et al. (2022) also observed ARGs and MGEs in both Day River water samples and shrimp ponds in Vietnam, where 11 ARGs have a significant correlation with both total MGEs and individual MGE types. The *Col* plasmid groups are often found to be associated with *E. coli* and *K. pneumonia*, having been found to disseminate AMR genes, which confers resistance to quinolones (Rozwandowicz et al., 2018; Khezri et al., 2021). The IncF plasmid types have been reported to harbor a wide range of resistance genes to aminoglycosides, tetracyclines, *β*-lactams, quinolones, and macrolides, reported from various systems such as riverine systems, wastewater treatment plants, drinking water sources. The *Inc*F plasmids often carry multiple replicons and harbor-reliant systems, which enable them to remain stable in bacterial host cells even under changing environmental conditions (Yang et al., 2015; Kesamang and Rahube, 2019; Ajayi et al., 2021). Additionally, the present study also identified 185 virulence factor genes (VFGs) from the river sediment metagenome, mostly from the BRS-2 sampling site. The metagenomic analysis uncovered ARGs along VFGs from different environments, such as river sediment, soil, animal fecal, etc. (Kim et al., 2022; Zou et al., 2023; Rout et al., 2024; Bai et al., 2024). Similar to the present study, Rout et al. (2024) investigated the prevalence of ARGs and VFGs within the sediment environment of the river Ganga, where a high diversity of VFGs includes *mshM* associated with adherence mechanisms; *pilH*, *pilT*, *pilU*, and *pilZ*, involved in twitching motility; such as *cheY*, *fliJ*, *fliQ,* and PA1464 genes were associated with chemotaxis and flagellar biosynthesis and *acpXL*, related to immune modulation. Bai et al. (2024) observed a total of 731 ARGs and 400 VFs common among humans, chickens, pigs, and soil environments, where a greater number of specific ARGs and VFGs were present in soil and chicken samples. The VFGs and plasmid-related genes were found to be highest in BRS-2 in sampling sites, which may be responsible for the observed higher ARG abundance (Jiang et al., 2022). Environmental variables, along with ARGs host influence the abundance of ARGs and VFGs by directly regulating MGEs and microbial community structure (Zou et al., 2023; Liu et al., 2024). The sites having high ARGs, MGEs-plasmid type, and VFGs are the sites along the Brahmaputra River passing through the major city areas, where human activities are involved, such as domestic waste, municipal waste, nearby hospital waste, transportation, etc.

In the network analysis, the co-occurrence pattern of ARGs was explored to illustrate the intricate relationships between various ARG subtypes. ARG subtypes assigned to the same ARG category (i.e., intratypes) usually co-occur. Li et al. (2015) noted the co-occurrence of environmental ARGs’ subtypes consisting of 46 nodes and 98 edges and highlighted the high prevalence of tetM and aminoglycoside resistance protein in different environments facilitating their applicability as ARG indicators. Gu et al. (2022) found a strong positive correlation among ARG subtypes, bacterial taxa, and mobile genetic elements, revealing a high ARG dissemination risk in drinking water treatment systems. Sang et al. (2023) described that the environmental factors greatly influenced the distribution of ARGs in the mangrove ecosystem, and the high abundance of ARGs was distributed in a modular manner. The presence of multidrug resistance genes in abundance and 100% occupancy of aminoglycoside-ARGs and β-lactam-ARGs in some of the sampling sites provides an overview of the role of human activities in accelerating spreading and proliferation of ARGs/VFGs in the urban river environment. It draws attention to the control of antibiotic use and emissions to protect public health. The presence of co-occurring ARGs suggests that these genes might be part of the same or closely related pathways, or they may be co-selected under antibiotic pressure. The co-occurrence of ARGs in environmental samples suggests that bacteria in these environments may be exposed to multiple selective pressures (e.g., antibiotic contamination, heavy metals, etc.). Co-occurrence patterns can enhance the horizontal gene transfer (HGT) potential among bacteria, increasing the spread of antibiotic resistance in the environment. These patterns are also of public health concern, as the spread of multidrug resistance could complicate the treatment of bacterial infections. This highlights the importance of monitoring and managing antibiotic usage and contamination in river environmental settings to mitigate the risk of resistance gene dissemination.

## Conclusion

By employing a metagenomic approach, the present study provides insights into microbiome community structure, its function, and the abundance of antimicrobial resistance genes in the sediments of selected stretches of the Brahmaputra River, India. The microbial diversity reveals a high abundance of Pseudomonadota at the phylum level, with *Bradyrhizobium*, *Pseudomonas*, and Var*iovorax* dominating at the genera level, while *T. denitrificans* emerges as the most abundant bacterial species across all sampling sites highlighting its crucial role in organic matter degradation and denitrification processes. The highest bacterial diversity and abundance were recorded upstream of the river, particularly at the initial point, in comparison to middle and downstream areas. Functional analysis revealed that the identified microbes exhibited diverse functional capabilities and adaptive strategies, with a significant portion involved in energy and amino acid metabolism, central carbon metabolism, stress response pathways, and degradation pathways. The presence of ARGs in the river indicated multiple drug-resistance genes linked to anthropogenic activities. The growing number of reports concerning antimicrobial resistance is alarming and posing challenges to global health initiatives. Since rivers such as the Brahmaputra are principal water sources for household, drinking, and personal hygiene, individuals using this water without prior treatment may experience serious health risks. To our knowledge, this is the first study to report the whole genome metagenomic sequencing of sediments in the Himalayan Brahmaputra River. Thus, it is essential to properly regulate the discharge of untreated sewage into the river. Consequently, the study emphasizes the need for further research on the important microbes and ARGs to better understand their interaction with the aquatic ecosystem and promote sustainable river health management.

## Data Availability

The datasets presented in this study can be found in online repositories. The names of the repository/repositories and accession number(s) can be found in the article/Supplementary material.
