# Supplementary material for: Metagenomic insights into microbial community, functional annotation, and antibiotic resistance genes in Himalayan Brahmaputra River sediment, India
Source: Front Microbiol. 2024 Nov 20;15:1426463. doi: 10.3389/fmicb.2024.1426463 (PMC11614985; doi:10.3389/fmicb.2024.1426463)
Supplement: Supplementary file 1 [file Supplementary_file_1.zip › Supplementary Table S1 and S2.DOCX]

**Table S1: Environmental factors of the water samples**

| **Environmental factors** | **BRS-1** | **BRS-2** | **BRS-3** | **BRS-4** | **BRS-5** | **BRS-6** |
| --- | --- | --- | --- | --- | --- | --- |
| Water temperature (°C) | 27.62 | 26.28 | 26.28 | 23.96 | 23.52 | 24.28 |
| pH | 7.7 | 7.84 | 7.76 | 7.72 | 7.95 | 7.93 |
| Dissolve Oxygen (ppm) | 7.824 | 6.694 | 6.782 | 7.774 | 8.438 | 7.864 |
| Alkalinity (ppm) | 76 | 75 | 82.6 | 70.6 | 67.8 | 86.2 |
| TDS (ppm) | 59.4 | 49 | 68.2 | 81.2 | 99.8 | 82.2 |
| Electrical Conductivity (uS/cm) | 88.6 | 88 | 92 | 110.6 | 76.4 | 100.2 |
| Salinity (ppt) | 0.01 | 0.01 | 0.01 | 0.034 | 0.014 | 0.012 |
| Turbidity (NTU) | 18.4 | 18.4 | 19.6 | 23.6 | 29.8 | 17.2 |
| Free CO_2_ (ppm) | 0.7 | 0.5 | 0.7 | 0.5 | 0.5 | 0.5 |
| Phosphate (ppm) | 0.003 | 0.005 | 0.012 | 0.009 | 0.016 | 0.016 |
| Nitrate (ppm) | 0.018 | 0.031 | 0.018 | 0.025 | 0.032 | 0.038 |

**Table S2: Environmental factors of the sediment samples**

| **Environmental factors** | **BRS-1** | **BRS-2** | **BRS-3** | **BRS-4** | **BRS-5** | **BRS-6** |
| --- | --- | --- | --- | --- | --- | --- |
| Soil texture | Sandy | Sandy | Sandy | Sandy | Sandy-loamy | Sandy-loamy |
| pH | 6.82 | 6.85 | 7.8 | 7.6 | 8.64 | 8.56 |
| Organic carbon (%) | 0.347 | 0.451 | 0.372 | 0.41 | 0.495 | 0.42 |
| Available P (mg/100 g sediment) | 0.52 | 0.57 | 0.61 | 0.54 | 0.38 | 0.45 |
| Available N(mg/100 g sediment) | 12 | 12 | 11.5 | 11.5 | 11 | 11 |
| Free CaCO_3_ (%) | 5 | 5 | 3.5 | 3.25 | 5 | 2.5 |
| Cr (ppm) | 5.5 | 6.45 | 7.56 | 8.49 | 7.06 | 5.54 |
| Mn (ppm) | 55.72 | 56.43 | 58.89 | 54.02 | 49.35 | 44.25 |
| Fe (ppm) | 2740.96 | 2789.71 | 3477.43 | 3328.03 | 3130.98 | 2466.45 |
| Cu (ppm) | 2.72 | 3.72 | 3.58 | 5.22 | 2.52 | 2.07 |
| Zn (ppm) | 5.84 | 8.61 | 10.48 | 12.83 | 0.52 | 0.52 |
| As (ppm) | 0.03 | 0.01 | 0.001 | 0.01 | 0.001 | 0.006 |
| Pb (ppm) | 5.7 | 5.55 | 8.29 | 8.97 | 3.57 | 4.52 |
