# Supplementary material for: Metagenomic insights into microbial community, functional annotation, and antibiotic resistance genes in Himalayan Brahmaputra River sediment, India
Source: Front Microbiol. 2024 Nov 20;15:1426463. doi: 10.3389/fmicb.2024.1426463 (PMC11614985; doi:10.3389/fmicb.2024.1426463)
Supplement: Supplementary file 1 [file Supplementary_file_1.zip › Supplementary Table S3.DOCX]

**Table S3: Details of all detected potential ARG sequences including contig name, start, end of hit, fasta sequences, similarity %, coverage % , etc. from sediment metagenome of River Brahmaputra from CARD database using Abricate tool (%COVERAGE >70; %IDENTITY >80)**

| **SAMPLING SITE: BRS-1** | | | | | | | | | | | |
| --- | --- | --- | --- | --- | --- | --- | --- | --- | --- | --- | --- |
| **SEQUENCE** | **START** | **END** | **STRAND** | **GENE** | **COVERAGE** | **COVERAGE_MAP** | **GAPS** | **%COVERAGE** | **%IDENTITY** | **ACCESSION** | **RESISTANCE** |
| **-** | **-** | **-** | **-** | **-** | **-** | **-** | **-** | **-** | **-** | **-** | **-** |

| **SAMPLING SITE: BRS-2** | | | | | | | | | | | |
| --- | --- | --- | --- | --- | --- | --- | --- | --- | --- | --- | --- |
| **SEQUENCE** | **START** | **END** | **STRAND** | **GENE** | **COVERAGE** | **COVERAGE_MAP** | **GAPS** | **%COVERAGE** | **%IDENTITY** | **ACCESSION** | **RESISTANCE** |
| k141_117004 | 1 | 1424 | - | mdsC | 1-1424/1512 | =============== | 0/0 | 94.18 | 98.45 | AE006468.2:393931-392419 | carbapenem;cephalosporin;cephamycin;monobactam;penam;penem;phenicol |
| k141_117004 | 1387 | 4554 | - | mdsB | 1-3168/3168 | =============== | 0/0 | 100 | 99.31 | AE006468.2:397061-393893 | carbapenem;cephalosporin;cephamycin;monobactam;penam;penem;phenicol |
| k141_117004 | 4551 | 5777 | - | mdsA | 1-1227/1227 | =============== | 0/0 | 100 | 98.78 | AE006468.2:398284-397057 | carbapenem;cephalosporin;cephamycin;monobactam;penam;penem;phenicol |
| k141_117004 | 8354 | 8818 | + | golS | 1-465/465 | =============== | 0/0 | 100 | 99.36 | AE006468.2:400860-401325 | carbapenem;cephalosporin;cephamycin;monobactam;penam;penem;phenicol |
| k141_120085 | 3288 | 4105 | - | bacA | 1-818/822 | ========/====== | 2-Feb | 99.39 | 85.84 | U00096.3:3204131-3203309 | peptide |
| k141_126504 | 2030 | 3486 | - | tolC | 1-1466/1488 | ========/====== | 2-Sep | 97.92 | 81.72 | FJ768952:0-1488 | aminocoumarin;aminoglycoside;carbapenem;cephalosporin;cephamycin;fluoroquinolone;glycylcycline;macrolide;penam;penem;peptide;phenicol;rifamycin;tetracycline;triclosan |
| k141_127183 | 248 | 1420 | + | emrA | 1-1173/1173 | ========/====== | 2-Feb | 99.91 | 82.28 | AP009048:2810082-2811255 | fluoroquinolone |
| k141_127183 | 1437 | 2975 | + | emrB | 1-1539/1539 | =============== | 0/0 | 100 | 84.28 | U00096:2812615-2814154 | fluoroquinolone |
| k141_131636 | 2816 | 3253 | - | AAC(6')-Iy | 1-438/438 | =============== | 0/0 | 100 | 98.4 | AF144880:3541-3979 | aminoglycoside |
| k141_133704 | 1 | 413 | - | emrR | 1-413/531 | ============... | 0/0 | 77.78 | 89.1 | U00096.3:2810769-2811300 | fluoroquinolone |
| k141_26364 | 4070 | 5263 | + | Escherichia_coli_acrA | 1-1194/1194 | =============== | 0/0 | 100 | 83.33 | U00096.3:485619-484425 | cephalosporin;fluoroquinolone;glycylcycline;penam;phenicol;rifamycin;tetracycline;triclosan |
| k141_2783 | 10085 | 10434 | + | marA | 1-350/384 | ==============. | 0/0 | 91.15 | 89.43 | AP009048.1:1621287-1621671 | carbapenem;cephalosporin;cephamycin;fluoroquinolone;glycylcycline;monobactam;penam;penem;phenicol;rifamycin;tetracycline;triclosan |
| k141_28044 | 34 | 706 | - | kdpE | 1-673/678 | ========/====== | 2-Feb | 99.12 | 81.6 | U00096.3:721733-721055 | aminoglycoside |
| k141_29221 | 335 | 1701 | - | cpxA | 1-1367/1374 | =============== | 0/0 | 99.49 | 85.08 | BA000007.3:4905062-4903688 | aminocoumarin;aminoglycoside |
| k141_31493 | 225 | 946 | - | baeR | 1-722/723 | =============== | 0/0 | 99.86 | 81.16 | AP009048.1:2166412-2167135 | aminocoumarin;aminoglycoside |
| k141_36134 | 630 | 1490 | + | TEM-116 | 1-861/861 | =============== | 0/0 | 100 | 100 | U36911.1:1429-2290 | cephalosporin;monobactam;penam;penem |
| k141_58435 | 178 | 591 | + | H-NS | 1-414/414 | =============== | 0/0 | 100 | 89.13 | BA000007.3:1738104-1737690 | cephalosporin;cephamycin;fluoroquinolone;macrolide;penam;tetracycline |
| k141_86664 | 1 | 646 | - | sul1 | 1-646/840 | ============... | 0/0 | 76.9 | 99.07 | JF969163:1053-1893 | sulfonamide |

| **SAMPLING SITE: BRS-3** | | | | | | | | | | | |
| --- | --- | --- | --- | --- | --- | --- | --- | --- | --- | --- | --- |
| **SEQUENCE** | **START** | **END** | **STRAND** | **GENE** | **COVERAGE** | **COVERAGE_MAP** | **GAPS** | **%COVERAGE** | **%IDENTITY** | **ACCESSION** | **RESISTANCE** |
| k141_111081 | 252 | 650 | + | emrR | 1-399/531 | ============... | 0/0 | 75.14 | 89.47 | U00096.3:2810769-2811300 | fluoroquinolone |
| k141_154369 | 4 | 514 | + | kdpE | 134-644/678 | ..======/====== | 2-Feb | 75.22 | 80.66 | U00096.3:721733-721055 | aminoglycoside |
| k141_177206 | 984 | 1821 | - | sul1 | 3-840/840 | =============== | 0/0 | 99.76 | 100 | JF969163:1053-1893 | sulfonamide |
| k141_177206 | 1907 | 2484 | - | aadA2 | 203-780/780 | ...============ | 0/0 | 74.1 | 100 | AF156486:5012-5792 | aminoglycoside |
| k141_38325 | 5 | 652 | - | bacA | 1-648/822 | ============... | 0/0 | 78.83 | 87.35 | U00096.3:3204131-3203309 | peptide |
| k141_61690 | 4 | 717 | - | baeR | 1-714/723 | =============== | 0/0 | 98.76 | 81.37 | AP009048.1:2166412-2167135 | aminocoumarin;aminoglycoside |
| k141_90613 | 138 | 998 | - | TEM-116 | 1-861/861 | =============== | 0/0 | 100 | 100 | U36911.1:1429-2290 | cephalosporin;monobactam;penam;penem |

| **SAMPLING SITE: BRS-4** | | | | | | | | | | | |
| --- | --- | --- | --- | --- | --- | --- | --- | --- | --- | --- | --- |
| **SEQUENCE** | **START** | **END** | **STRAND** | **GENE** | **COVERAGE** | **COVERAGE_MAP** | **GAPS** | **%COVERAGE** | **%IDENTITY** | **ACCESSION** | **RESISTANCE** |
| k141_203123 | 9 | 872 | - | mdtH | 1-864/1209 | ===========.... | 0/0 | 71.46 | 81.02 | U00096:1125326-1124117 | fluoroquinolone |
| k141_53707 | 565 | 978 | + | H-NS | 1-414/414 | =============== | 0/0 | 100 | 88.89 | BA000007.3:1738104-1737690 | cephalosporin;cephamycin;fluoroquinolone;macrolide;penam;tetracycline |
| k141_55689 | 559 | 1419 | + | TEM-116 | 1-861/861 | =============== | 0/0 | 100 | 100 | U36911.1:1429-2290 | cephalosporin;monobactam;penam;penem |
| k141_75863 | 113 | 745 | + | CRP | 1-633/633 | =============== | 0/0 | 100 | 87.99 | AP009048.1:4154296-4153663 | fluoroquinolone;macrolide;penam |
| k141_90002 | 297 | 1523 | - | mdsA | 1-1227/1227 | =============== | 0/0 | 100 | 98.53 | AE006468.2:398284-397057 | carbapenem;cephalosporin;cephamycin;monobactam;penam;penem;phenicol |
| k141_93607 | 66 | 596 | + | emrR | 1-531/531 | =============== | 0/0 | 100 | 85.88 | U00096.3:2810769-2811300 | fluoroquinolone |

| **SAMPLING SITE: BRS-5** | | | | | | | | | | | |
| --- | --- | --- | --- | --- | --- | --- | --- | --- | --- | --- | --- |
| **SEQUENCE** | **START** | **END** | **STRAND** | **GENE** | **COVERAGE** | **COVERAGE_MAP** | **GAPS** | **%COVERAGE** | **%IDENTITY** | **ACCESSION** | **RESISTANCE** |
| k141_129407 | 1 | 1159 | + | mdsA | 69-1227/1227 | =============== | 0/0 | 94.46 | 98.62 | AE006468.2:398284-397057 | carbapenem;cephalosporin;cephamycin;monobactam;penam;penem;phenicol |
| k141_15560 | 98 | 562 | - | golS | 1-465/465 | =============== | 0/0 | 100 | 99.14 | AE006468.2:400860-401325 | carbapenem;cephalosporin;cephamycin;monobactam;penam;penem;phenicol |
| k141_174328 | 362 | 1449 | + | cpxA | 1-1088/1374 | ============... | 0/0 | 79.18 | 85.94 | BA000007.3:4905062-4903688 | aminocoumarin;aminoglycoside |
| k141_183481 | 336 | 718 | - | AAC(6')-Iaa | 56-438/438 | .============== | 0/0 | 87.44 | 96.34 | AE006468.2:1707351-1707789 | aminoglycoside |
| k141_195753 | 250 | 1110 | - | TEM-116 | 1-861/861 | =============== | 0/0 | 100 | 100 | U36911.1:1429-2290 | cephalosporin;monobactam;penam;penem |
| k141_209683 | 172 | 667 | - | kdpE | 178-673/678 | ...=====/====== | 2-Feb | 73.01 | 80.89 | U00096.3:721733-721055 | aminoglycoside |
| k141_2662 | 1 | 626 | - | sul1 | 1-626/840 | ============... | 0/0 | 74.52 | 100 | JF969163:1053-1893 | sulfonamide |
| k141_27370 | 48 | 1114 | + | mdtK | 1-1067/1425 | ============... | 0/0 | 74.88 | 98.78 | CP014358.1:2162750-2161325 | fluoroquinolone |
| k141_40624 | 1 | 403 | + | emrR | 69-471/531 | .=============. | 0/0 | 75.89 | 86.6 | U00096.3:2810769-2811300 | fluoroquinolone |
| k141_62260 | 1678 | 2032 | - | ramA | 21-375/375 | =============== | 0/0 | 94.67 | 80 | JQ727668:0-375 | carbapenem;cephalosporin;cephamycin;fluoroquinolone;glycylcycline;monobactam;penam;penem;phenicol;rifamycin;tetracycline;triclosan |
| k141_68648 | 158 | 507 | + | marA | 1-350/384 | ==============. | 0/0 | 91.15 | 89.14 | AP009048.1:1621287-1621671 | carbapenem;cephalosporin;cephamycin;fluoroquinolone;glycylcycline;monobactam;penam;penem;phenicol;rifamycin;tetracycline;triclosan |
| k141_68789 | 113 | 526 | + | H-NS | 1-414/414 | =============== | 0/0 | 100 | 88.89 | BA000007.3:1738104-1737690 | cephalosporin;cephamycin;fluoroquinolone;macrolide;penam;tetracycline |
| k141_8807 | 418 | 1929 | + | mdsC | 1-1512/1512 | =============== | 0/0 | 100 | 97.75 | AE006468.2:393931-392419 | carbapenem;cephalosporin;cephamycin;monobactam;penam;penem;phenicol |
| k141_92487 | 1 | 909 | + | emrA | 265-1173/1173 | ...============ | 0/0 | 77.49 | 81.85 | AP009048:2810082-2811255 | fluoroquinolone |

| **SAMPLING SITE: BRS-6** | | | | | | | | | | | |
| --- | --- | --- | --- | --- | --- | --- | --- | --- | --- | --- | --- |
| **SEQUENCE** | **START** | **END** | **STRAND** | **GENE** | **COVERAGE** | **COVERAGE_MAP** | **GAPS** | **%COVERAGE** | **%IDENTITY** | **ACCESSION** | **RESISTANCE** |
| k141_155341 | 107 | 924 | + | bacA | 1-818/822 | ========/====== | 2-Feb | 99.39 | 85.47 | U00096.3:3204131-3203309 | peptide |
| k141_382980 | 1 | 621 | - | TEM-116 | 1-621/861 | ===========.... | 0/0 | 72.13 | 99.84 | U36911.1:1429-2290 | cephalosporin;monobactam;penam;penem |
| k141_50139 | 1 | 352 | + | H-NS | 63-414/414 | ..============= | 0/0 | 85.02 | 86.93 | BA000007.3:1738104-1737690 | cephalosporin;cephamycin;fluoroquinolone;macrolide;penam;tetracycline |
| k141_8591 | 178 | 532 | + | ramA | 21-375/375 | =============== | 0/0 | 94.67 | 79.72 | JQ727668:0-375 | carbapenem;cephalosporin;cephamycin;fluoroquinolone;glycylcycline;monobactam;penam;penem;phenicol;rifamycin;tetracycline;triclosan |
