# Supplementary material for: Metagenomic insights into microbial community, functional annotation, and antibiotic resistance genes in Himalayan Brahmaputra River sediment, India
Source: Front Microbiol. 2024 Nov 20;15:1426463. doi: 10.3389/fmicb.2024.1426463 (PMC11614985; doi:10.3389/fmicb.2024.1426463)
Supplement: Supplementary file 1 [file Supplementary_file_1.zip › Supplementary Table S4.DOCX]

| **Table S4: Abundance of Virulence factor genes (VFGs) from the sediment metagenome of the six sampling sites (BRS-1 to BRS-6) in River Brahmaputra, India obtained from Virulence factor database using Abricate tool** | | | | | | |
| --- | --- | --- | --- | --- | --- | --- |
| **SAMPLING SITES** | **BRS-6** | **BRS-2** | **BRS-4** | **BRS-1** | **BRS-3** | **BRS-5** |
| **Total VFGs** | **37** | **132** | **68** | **13** | **56** | **92** |
| *IlpA* | 87.41 | 0 | 0 | 0 | 0 | 0 |
| *acpXL* | 93.26 | 96.45 | 90.07;93.25;79.32;81.86;94.94;80.59;96.81;73.05;94.94 | 88.61;82.28;94.94;81.01;92.41 | 93.25;96.20 | 88.61;88.61;79.75;94.94 |
| *alg8* | 0 | 0 | 0 | 0 | 0 | 89.29;88.62 |
| *algA* | 0 | 0 | 0 | 0 | 93.36;88.45 | 93.08;94.33;95.09 |
| *algB* | 0 | 0 | 0 | 0 | 0 | 95.63 |
| *algD* | 0 | 0 | 0 | 0 | 82.68 | 99.54;86.19 |
| *algF* | 0 | 0 | 0 | 0 | 81.41 | . |
| *algI* | 0 | 0 | 0 | 0 | 0 | 74.98 |
| *algJ* | 0 | 0 | 76.96 | 0 | 76.96 | 77.21 |
| *algL* | 0 | 0 | 0 | 0 | 0 | 78.71 |
| *algR* | 0 | 0 | 77.91 | 0 | 77.91 | 0 |
| *algU* | 90.21 | 78.87 | 86.6 | 0 | 70.79;89.35;82.65 | 89.69 |
| *avrA* | 0 | 100 | 0 | 0 | 0 | 0 |
| *bplA* | 0 | 0 | 81.58 | 0 | 0 | 0 |
| *bplB* | 0 | 0 | 71.35 | 0 | 78.99 | 0 |
| *cheB* | 89.22 | 96.67 | 89.31 | 73.37 | 87.32 | 89.22 |
| *cheR* | 82.38 | 79.43 | 96.86 | . | 74.89 | 74.89 |
| *cheW* | 79.92 | 84.28;75.19 | 89.02;76.14;84.09;74.24;84.28;71.78;78.98 | . | 70.45;75.00;84.09;78.22 | 84.28 |
| *cheY* | 86.41 | 83.68;81.79 | 84.62;71.79;84.10;87.88;84.38;99.49;92.82 | 84.85 | 99.49 | 99.49 |
| *cheZ* | . | 87.29 | 0 | 0 | 0 | 0 |
| *clpP* | . | 0 | 0 | 93.97 | 0 | 0 |
| *clpV1* | . | 0 | 0 | 0 | 94.24 | 94.5 |
| *csgA* | 85.53 | 100 | 100 | 0 | 85.53 | 0 |
| *csgB* | . | 100 | 0 | 0 | 0 | 100 |
| *csgC* | . | 96.94 | . | 0 | 0 | 100 |
| *csgD* | . | 100 | . | 0 | 0 | 0 |
| *csgE* | . | 100 | 100 | 0 | 77.27 | 0 |
| *csgF* | . | 100 | . | 0 | 0 | 0 |
| *csgG* | . | 100 | . | 0 | 73.14 | 0 |
| *ddhA* | . | 98.35 | 81.3 | 0 | 0 | 81.81 |
| *ddhB* | . | 88.18 | . | . | . | . |
| *dotU1* | . | . | . | . | . | 71.11 |
| *entB* | . | 99.18 | 99.18 | . | 71.33 | 99.18 |
| *entC* | . | 99.75 | . | . | . | 88.3 |
| *entE* | . | 98.82 | . | . | . | . |
| *entS* | . | 98.08 | 98.08 | . | . | 78.02 |
| *faeC* | . | 91.58 | . | . | . | 82.97 |
| *faeH* | . | 79.57 | . | . | . | . |
| *faeI* | . | 98.04 | . | . | . | . |
| *faeJ* | . | . | . | . | . | 82.17 |
| *fepA* | . | 99.06 | . | . | . | . |
| *fepB* | . | . | 84.01 | . | . | . |
| *fepC* | . | 79.53 | . | . | . | . |
| *fepD* | . | 98.53 | 87.91 | . | 87.12 | . |
| *fepG* | . | 83.28 | . | . | . | . |
| *fes* | . | 96.92 | . | . | . | . |
| *fimC* | . | 100 | . | . | . | . |
| *fimF* | . | . | . | . | . | 100 |
| *fimH* | . | 100 | . | . | . | . |
| *fimI* | . | 100 | 100 | . | . | . |
| *fleN* | . | . | 93 | . | 92.05 | . |
| *flgB* | 74.75 | . | . | . | . | . |
| *flgC* | 90.86;80.28;88.26;87.79 | 84.04;70.66;96.79 | 73.24;88.03;88.26;88.26 | 73.71 | 72.07;76.76;96.79 | 80.75;96.79 |
| *flgF* | . | 99.47 | 86.61 | . | . | 99.47 |
| *flgG* | 70.34;94.04 | 99.69;92.52;82.38 | 92.75;72.88;89.23;95.94;81.37;94.04 | . | 99.69;92.75;86.06;89.23 | 99.69;94.04;95.17;93.16 |
| *flgH* | . | 80.85 | 79.68 | . | . | . |
| *flgI* | . | 95.73 | 89.46;86.52 | 82.16 | 96.49 | 93.15;93.06 |
| *flhA* | . | 99.28 | . | . | . | 73.66;75.47 |
| *flhB* | . | 96.01 | . | . | . | 86.19 |
| *flhC* | . | 95.19 | . | . | . | . |
| *flhD* | . | 78.06 | . | . | . | 78.06 |
| *fliA* | 81.28;84.84;89.89 | 75.41;82.10;95.71;80.05;89.48;89.48 | 74.04;84.43 | 76.37;75.96 | 84.43;75.55;84.54 | 84.43;80.74;85.62;83.60;81.01;82.10 |
| *fliG* | 92.72;91.87 | 96.89;96.37;95.08 | 75.40;79.12 | . | 87.65 | 77.41;94.78;92.63;93.22;88.76 |
| *fliI* | . | 92.57 | . | . | . | 72.01 |
| *fliM* | 74.87;92.29 | 99.90;91.49 | 75.38;71.77;92.29;81.88;73.46 | . | 91.59 | 87.65;74.79;77.08;76.98;83.78 |
| *fliP* | 79.56;80.47;84.57;74.80 | 82.02;95.05 | 79.56;71.61;70.57;86.88;74.48 | . | 73.75 | 87.01;71.26;86.61;88.94 |
| *fliQ* | 91.48 | . | 73.26;84.98;82.78;71.06 | . | . | 76.56;84.62;76.56;84.62;91.48 |
| *fliS* | . | 83.71 | . | . | . | 83.71 |
| *galU* | . | 91.78 | . | . | . | . |
| *gmd* | 90.08 | . | 72.73 | . | 78.42 | 75.76 |
| *gmhA/lpcA* | . | 70.6 | . | . | . | 70.6 |
| *gtrA* | 100 | 100 | 100 | . | . | 75.21 |
| *hasC* | . | . | . | 72.13;85.79 | . | . |
| *hsiB1/vipA* | 83.62;72.25 | . | 90.56;89.02 | . | . | 89.02 |
| *hsiC1/vipB* | . | . | . | . | 95.19 | 95.19 |
| *htpB* | 75.2 | 86.15 | 97.7 | 75.14 | 97.7 | . |
| *icl* | 73.5 | . | 74.67 | . | . | 95.26 |
| *invA* | . | 100 | . | . | . | . |
| *invB* | . | 100 | 78.19 | . | . | 100 |
| *invE* | . | . | 72.21 | . | . | . |
| *invF* | . | 100 | . | . | . | . |
| *invH* | . | 100 | . | . | . | . |
| *invI* | . | 100 | 91.67 | . | . | 98.2 |
| *invJ* | . | 100 | . | . | . | 98.02 |
| *iroB* | . | 95.88 | . | . | . | . |
| *iroC* | . | 96.47 | . | . | . | . |
| *iroD* | . | 94.31 | 86.02 | . | . | . |
| *iroE* | . | 85.37 | . | . | . | 75.65 |
| *iroN* | . | 98.94 | . | . | . | . |
| *katA* | . | . | 70.69 | . | . | 82.18 |
| *katB* | . | . | 71.99 | . | . | 93.44 |
| *kdsA* | 95.2 | . | 90.88 | . | . | . |
| *kdsB* | . | . | 72.97 | . | . | . |
| *lpeA* | . | . | . | 88.21 | . | . |
| *lpfA* | . | 100 | . | . | . | 100 |
| *lpfB* | . | 80.83 | 93.42 | . | . | 100 |
| *lpfC* | . | 100 | . | . | . | . |
| *lpfE* | 89.77 | 100 | 88.64 | . | . | . |
| *lpxB* | . | 78.52 | . | . | . | 78.6 |
| *lpxC* | . | 93.14 | 71.68 | . | . | 93.14 |
| *luxS* | 80.15 | . | . | . | . | 80.15 |
| *mgtC* | . | 100 | . | . | . | 87.07 |
| *mig-14* | . | 100 | . | . | . | . |
| *misL* | . | 71.16 | . | . | . | . |
| *motA* | 92.57;85.02;97.10 | 93.24;71.20 | 97.10;92.92 | . | 73.05;73.52;89.55 | 94.25;97.10 |
| *motC* | . | . | . | . | 80.97;98.92 | 99.06;98.92 |
| *ompA* | . | 78.19 | . | . | . | . |
| *orgA* | . | 100 | . | . | . | 84.83 |
| *orgB* | . | 100 | . | . | 73.86 | . |
| *orgC* | . | . | 100 | . | . | . |
| *pefA* | . | 71.1 | . | . | . | . |
| *pgm* | 72.19 | . | 71.9 | . | 71.96 | 86.01;83.95 |
| *pilC* | . | . | 71.11;98.84 | . | 95.29 | 95.29;88.36 |
| *pilG* | 80.15;88.73;85.78;83.58;85.78;85.54;78.19;86.76;77.70;82.60;75.00;91.67 | 78.68;78.43;78.43 | 88.73;72.55;78.19;77.45;81.86;71.08;71.08;78.43;79.66;81.62;78.19;85.05;81.37 | 80.88 | 78.43;78.19;78.19;78.68;88.73;91.42;77.70;81.86 | 78.43;81.62;79.66;82.11;80.88 |
| *pilH* | 87.98;81.69;72.68;75.14;85.79 | 70.49 | 87.98;88.25;72.95 | 81.15 | 70.22;87.98;88.25;82.79 | 90.98;77.05;75.14 |
| *pilM* | . | . | 95.77;83.19 | . | 95.77 | . |
| *pilP* | 86.1 | . | . | . | . | . |
| *pilR* | . | . | . | . | 78.33 | 78.92;97.23 |
| *pilT* | 82.18;81.74;98.84;73.24 | 80.84;91.50 | 88.21;85.31;82.18;72.89;80.97;91.01;71.21;85.82;77.68 | . | 71.59;99.90;88.89;98.55 | 95.27;94.01;79.50;98.55 |
| *pilU* | 74.33;89.56;92.34 | 74.06 | 70.58;73.89;70.50;73.28 | . | 92.34 | 85.73;89.47;87.73;87.55 |
| *pilW* | . | 75.68 | . | . | . | . |
| *pipB* | . | 100 | . | . | . | . |
| *pipB2* | . | 100 | . | . | . | . |
| *pla* | . | 84.03 | . | . | . | 84.03 |
| *prgH* | . | 100 | . | . | . | . |
| *prgI* | . | 100 | 85.19 | . | . | 100 |
| *prgJ* | 100 | 100 | . | . | . | 100 |
| *prgK* | . | 100 | . | . | . | . |
| *rck* | . | . | 79.03 | . | . | . |
| *rfaD* | . | 98.6 | 98.6 | . | . | . |
| *rfaE* | . | 77.64 | . | . | . | . |
| *rffG* | . | 75.05 | . | . | . | . |
| *sicA* | . | 100 | 100 | . | . | 99.2 |
| *sicP* | . | 100 | . | . | . | 100 |
| *sifA* | . | 89.02 | . | . | . | . |
| *sifB* | . | 95.06 | . | . | . | . |
| *sipA/sspA* | . | 80.76 | . | . | . | . |
| *sipB/sspB* | . | . | 91.02 | . | . | . |
| *sipC/sspC* | . | 100 | . | . | . | . |
| *sipD* | . | 100 | . | . | . | . |
| *sodCI* | . | 100 | . | . | . | 96.25 |
| *sopB/sigD* | . | 81.85 | . | . | . | . |
| *sopD* | . | 100 | . | . | . | . |
| *sopD2* | . | 74.17 | . | . | . | . |
| *sopE2* | . | 93.22 | . | . | . | . |
| *spaO* | . | 99.89 | . | . | 88.05 | . |
| *spaP* | . | . | . | . | . | 92.44 |
| *spaQ* | . | 100 | . | . | . | . |
| *spaR* | . | 100 | 97.73 | . | . | 78.66 |
| *spaS* | . | 100 | . | . | . | . |
| *spiC/ssaB* | . | 100 | . | . | 100 | 83.08 |
| *sptP* | . | . | . | . | 83.88 | . |
| *ssaE* | . | 100 | 100 | . | 72.84 | 100 |
| *ssaG* | . | 100 | . | . | . | . |
| *ssaH* | . | 100 | . | . | . | . |
| *ssaI* | . | 100 | 96.79 | . | . | . |
| *ssaJ* | . | 100 | . | . | . | . |
| *ssaK* | . | 100 | . | . | . | 73.48 |
| *ssaM* | . | 100 | 100 | . | . | . |
| *ssaN* | . | . | . | . | 77.88 | 100 |
| *ssaO* | . | 100 | . | . | 77.25 | . |
| *ssaP* | . | 100 | . | . | . | 100 |
| *ssaQ* | . | 73.68 | . | . | . | . |
| *ssaR* | . | 100 | . | . | . | . |
| *ssaT* | . | 81.67 | 95 | . | . | . |
| *sscA* | . | 100 | . | . | 100 | 94.94 |
| *sscB* | . | 87.36 | . | . | 100 | . |
| *sseA* | . | . | . | . | 97.86 | . |
| *sseB* | . | 100 | . | . | . | 97.97 |
| *sseC* | . | 100 | . | . | . | . |
| *sseD* | . | 87.24 | . | . | . | 75.34 |
| *sseE* | . | 100 | . | . | . | 100 |
| *sseF* | . | 99.87 | . | . | . | . |
| *sseG* | . | 100 | . | . | . | . |
| *sseK1* | . | 100 | . | . | . | . |
| *sseL* | . | . | . | . | . | 100 |
| *steA* | . | 81.83 | . | . | . | 80.25 |
| *steB* | . | 100 | . | . | 100 | . |
| *ureG* | 73.50;70.83;71.67 | . | . | 84.17 | . | . |
| *waaA* | . | . | . | . | 80.52 | 78.56 |
| *waaF* | 86.13 | . | 89.4 | . | 86.13 | 86.13 |
| *xcpA/pilD* | 75.03;86.71 | . | 85.57;75.37 | . | 90.15;82.25;73.65 | 89.00;90.49;70.45;81.90 |
| *xcpR* | . | . | 85.49 | . | 85.49 | 76.61;81.11 |
| *xcpS* | . | . | . | . | . | 75.62;94.25 |
| *xcpT* | 79.64;85.01 | 86.35;86.58;87.02 | 89.26;88.59;87.25;85.01 | . | 85.01;85.46;88.59 | 88.59;82.77;86.35;88.59 |
