# Supplementary material for: Metagenomic insights into microbial community, functional annotation, and antibiotic resistance genes in Himalayan Brahmaputra River sediment, India
Source: Front Microbiol. 2024 Nov 20;15:1426463. doi: 10.3389/fmicb.2024.1426463 (PMC11614985; doi:10.3389/fmicb.2024.1426463)
Supplement: Supplementary file 1 [file Supplementary_file_1.zip › Supplementary Figure S1.doc]

**Fig. S1.** CCA of the relative abundance of the microbiome at the phylum level with water quality parameters in the six samples of river Brahmaputra. Black color dot represents the objects [e.g. sampling sites (BRS-1 to BRS-6)].Blue color dot represents the response variables (e.g. microbial phylum & water parameters). Green lines represents quantitative explanatory variables (e.g. water parameters) with long lines indicating their direction of increase.
